# The influence of error detection and error significance on neural and behavioral correlates of error processing in a complex choice task

**DOI:** 10.3758/s13415-022-01028-6

**Published:** 2022-08-02

**Authors:** Elisa Porth, André Mattes, Jutta Stahl

**Affiliations:** Department of Individual Differences and Psychological Assessment, University of Cologne, Pohligstraße 1, 50969 Köln, Germany

**Keywords:** Error detection, Error significance, Self-evaluation, Task complexity, Event-related potential, Multivariate pattern analysis

## Abstract

Error detection and error significance form essential mechanisms that influence error processing and action adaptation. Error detection often is assessed by an immediate self-evaluation of accuracy. Our study used cognitive neuroscience methods to elucidate whether self-evaluation itself influences error processing by increasing error significance in the context of a complex response selection process. In a novel eight-alternative response task, our participants responded to eight symbol stimuli with eight different response keys and a specific stimulus-response assignment. In the first part of the experiment, the participants merely performed the task. In the second part, they also evaluated their response accuracy on each trial. We replicated variations in early and later stages of error processing and action adaptation as a function of error detection. The additional self-evaluation enhanced error processing on later stages, probably reflecting error evidence accumulation, whereas earlier error monitoring processes were not amplified. Implementing multivariate pattern analysis revealed that self-evaluation influenced brain activity patterns preceding and following the response onset, independent of response accuracy. The classifier successfully differentiated between responses from the self- and the no-self-evaluation condition several hundred milliseconds before response onset. Subsequent exploratory analyses indicated that both self-evaluation and the time on task contributed to these differences in brain activity patterns. This suggests that in addition to its effect on error processing, self-evaluation in a complex choice task seems to have an influence on early and general processing mechanisms (e.g., the quality of attention and stimulus encoding), which is amplified by the time on task.

**Supplementary Information:**

The online version contains supplementary material available at 10.3758/s13415-022-01028-6.

## Introduction

Open error culture gives the advice that errors exist to be learned from. We also know that dealing with errors is essential in terms of changing the behavior towards an intended target state (Wessel, 2018). Unfortunately, we still do not fully understand how errors are processed by the brain. The extent to which error processing and the subsequent adaptation of behavior take place varies as a function of several variables, with error detection and error significance being key influences. Error detection is usually investigated by an immediate self-evaluation of response accuracy. Based on that, errors can then be divided into errors that the actor signals as such (i.e., signaled errors) and errors that the actor does not signal as such (i.e., non-signaled errors; Maier et al., 2010; Stahl et al., 2020). Several studies have shown that signaled errors do not differ from non-signaled errors in earlier stages of error processing (Nieuwenhuis et al., 2001). However, in later stages of processing, signaled errors seem to be marked by more error evidence accumulation (Steinhauser & Yeung, 2010), which leads to error awareness (Nieuwenhuis et al., 2001). On a neural level, an earlier stage of error processing is represented by a component of the event-related potential (ERP), the error-related negativity (ERN, Gehring et al., 1993), also termed error negativity (N_e_, Falkenstein et al., 1991), which peaks between 0 and 150 ms after an erroneous response and is increased for signaled and non-signaled errors compared with correct responses (correct response negativity/correct negativity [CRN/N_c_]) (Falkenstein et al., 1991). A later stage is reflected by the error positivity (P_e_, peaking between 150 and 300 ms after an erroneous response) (Falkenstein et al., 1991; Endrass et al., 2007; Overbeek et al., 2005, Steinhauser & Yeung, 2010; Steinhauser & Yeung, 2012), which is increased for signaled errors compared with non-signaled errors and correct responses (Nieuwenhuis et al., 2001). Adaptational control processes often take place after signaled errors, such as in the form of post-error slowing (Laming, 1968; Rabbitt & Rodgers, 1977), while they are less frequently found after non-signaled errors (Klein et al., 2007; Nieuwenhuis et al., 2001; Wessel et al., 2011).

The multifaceted influence of error detection on cognitive processes underlines the importance of a profound understanding of this key variable. To assess error detection, tasks often employ an immediate self-evaluation of response accuracy after each trial. However, this additional task (i.e., the self-evaluation) might modulate error processing itself by enhancing the perceived significance of errors and the attention directed towards them. Previous studies have shown that varying error significance impacts the different stages of error processing, behavioral performance and adaptation mechanisms (Ullsperger & von Cramon, 2004). In a two-choice flanker task, Grützmann et al. (2014) investigated whether the implementation of a response accuracy rating influences error processing. They assumed that if an additional self-evaluation of accuracy enhanced error significance (there error salience) or the attention directed towards errors, this should translate to an increase of N_e_ amplitude. Alternatively, the authors argued that the attentional resources needed for the additional self-evaluation might lead to a reduction of available resources for the primary task and thus to a decreased N_e_ amplitude. Grützmann et al. (2014) found an increase in N_e_ and P_e_ amplitude when participants were asked to rate their response accuracy, delivering first evidence that self-evaluation might enhance error significance. Other studies delivered similar evidence by showing that early error processing was more pronounced when monetary incentives for correct responses were high, and therefore errors were perceived as more severe, which was reflected by a larger N_e_ amplitude (Ganushchak & Schiller, 2008; Hajcak et al., 2005; Hsieh et al., 2010; Maruo et al., 2016; Pailing & Segalowitz, 2004; Ullsperger & von Cramon, 2004). Maier and colleagues also supported the error significance account in various studies using the Eriksen-Flanker-Task (Maier et al., 2008, 2011, 2012; Maier & Steinhauser, 2016). However, other studies did not find evidence for this account (Maruo et al., 2017; Olvet & Hajcak, 2009b; Paul et al., 2017) and thus challenged the idea of error significance impacting the N_e_.

The error evidence accumulation process, reflected by the P_e/c_, also might be affected by error significance in terms of mood modulation (Paul et al., 2017) and monetary punishment (Maruo et al., 2016,  2017). Interestingly, the effects of error significance were not necessarily reflected in the participants’ performance (*response time*: Ganushchak & Schiller, 2008; Hajcak et al., 2005; Maruo et al., 2016; Paul et al., 2017; *accuracy*: Ganushchak & Schiller, 2008; Hajcak et al., 2005; Paul et al., 2017; Riesel et al., 2012; *post-error slowing*: Paul et al., 2017), although a few studies did report such influences (Grützmann et al., 2014; Hsieh et al., 2010; Maruo et al., 2016; Olvet & Hajcak, 2009b; Riesel et al., 2012). The listed studies overall support the idea that self-evaluation enhances error processing by increasing error significance and the attention toward error processing. However, the majority of the reported studies have implemented response tasks with a binary response mapping (e.g., left-hand and right-hand index finger), which keeps their findings distant from more complex everyday actions. To overcome this limitation, we have developed an eight-alternative response task (8ART; Stahl et al., 2020) that induces a higher cognitive load during response selection. In this task, we provide evidence for variations in neural and behavioral correlates of error processing with error detection given a more complex response selection (Stahl et al., 2020).

In the current study, we used a modified version of the 8ART, which has allowed us to gain further insight into these variations. Using tasks with different characteristics and increasing task complexity are important steps in investigating self-evaluation and error processing to elucidate whether the mechanisms are similar for different task requirements and whether different electrophysiological and behavioral markers might provide evidence for variations in these mechanisms (Olvet & Hajcak, 2009b ; Riesel et al., 2015). We added an immediate self-evaluation of response accuracy in the 8ART (Stahl et al., 2020) after each trial as a tool to assess error detection and confidence consecutively. Considering the effects of error significance on error processing and the promising results delivered by Grützmann et al. (2014), the question arises whether or not self-evaluation enhances error processing in a task with a more complex response selection and a higher cognitive load.

### Study objectives

Given that we used a modified version of the 8ART (Stahl et al., 2020) to investigate self-evaluation effects, our first goal was to examine general error detection effects on the neural and the behavioral correlates of error processing to gain further insight into error processing in more complex response tasks. Our second goal was to elucidate whether in our more complex task, we were able to replicate that the presence or absence of a self-evaluation in terms of response accuracy modified error processing and participants’ performance. While participants simply performed the 8ART in the first part of the experiment (i.e., without self-evaluation), they also had to evaluate their response accuracy on each trial in the second part. If self-evaluation enhances error significance or the attention directed towards errors, then the N_e_ and P_e_ amplitudes should be larger for errors that occur when a self-evaluation is required compared to errors after which no self-evaluation is required. If not, then no differences in N_e_ and P_e_ amplitudes should be observed between the two conditions. The question of whether or not this effect translates to the participants’ performance needs to be explored.

Because univariate ERP analyses did not show stable N_e/c_ effects under previous task settings (only under specific conditions, see Stahl et al., 2020), we further wanted to use a more sensitive method—a machine learning based approach (for details see *Methods*)—to be able to identify even small differences between the self-evaluation and the no-self-evaluation condition, and their onset if any existed. Based on this approach, Bode and Stahl (2014) have already shown differences between errors and correct responses starting approximately 90 ms before response onset. In the present study, we wanted to investigate whether we were able to obtain similar findings with the 8ART where response selection is much more challenging and whether there were differences between the two evaluation conditions.

## Methods

### Participants

A total of 40 participants, all undergraduate psychology students, took part in the experiment. For the assessment of error processing as a function of error detection (first research goal), the responses were categorized into signaled and non-signaled. Against expectations, only 21 participants (4 males, 17 females, none diverse; age: *M* = 22.4 years, *SD* = 4.5 years) produced enough non-signaled error trials (more than 6; see Olvet & Hajcak, 2009a, for details see limitations). With this sample size, a Type-I error of 0.05, and a power of 0.80, we were able to uncover effect sizes of η_p_^2^ ≥ 0.14 (G*Power; Faul et al., 2009). To assess the influence of self-evaluation on error significance (second research goal), it was sufficient to categorize the responses into correct and erroneous. Hence, only seven participants from the entire sample had to be excluded from analyses due to an insufficient number of errors trials (*n* = 6) and an experimenter error (*n* = 1, data saving for EEG was started too late), which led to a final sample size of 33 participants (7 males, 26 females, none diverse; age: *M* = 22.2 years, *SD* = 4.1 years). With this sample size, a Type-I error of 0.05 and a power of 0.80, we were able to uncover effect sizes of η_p_^2^ ≥ 0.11 (G*Power; Faul et al., 2009). The participants were recruited via a web-based recruitment system (Gotzhein & Elson, 2020), and they received course credit as a reward for participation. All of the participants reported normal or corrected-to-normal vision. Informed, written consent was obtained from each participant. This study was approved by the ethics committee of the German Psychological Association.

### Procedure

**Fig. 1:**
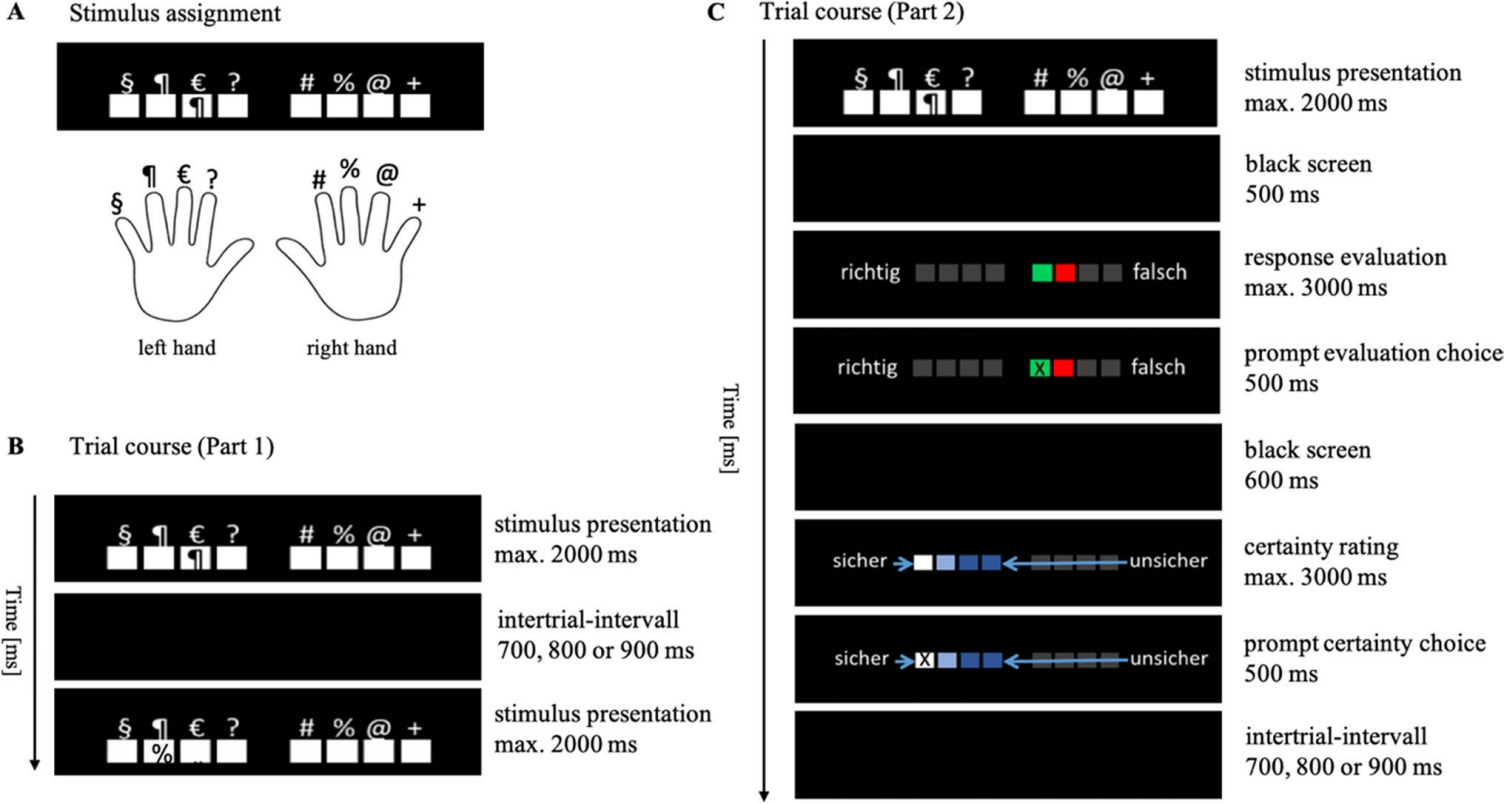
Assignment of the symbol stimuli to participant’s fingers (**a**), the course of one trial from part 1 (**b**), and the course of one trial from part 2, including response evaluation and certainty rating (**c**)

The participants completed the 8ART. On each trial, eight white squares appeared on the screen (TFT, 22”) in front of a black background. A different symbol was located above each of the eight squares (from left to right: §, ¶, &, ?, #, %, @, +). In every trial, one of the eight symbols also appeared inside one of the white squares. The participants responded to the symbol inside the white square by pressing one of eight force-sensitive response keys. The keys were positioned in front of the participants. The participants placed their fingers, excluding their thumbs, onto the keys. Each key, and therefore each finger, was assigned to one of the eight symbols above the white squares on the screen. The assignment of the symbols to the participant’s fingers is depicted in Fig. 1.

The participants were instructed to respond to the symbol appearing in one of the squares with the respective finger that the symbol was assigned to and they were told to ignore the position of the appearing symbol. They were further instructed to do so as fast and as accurately as possible. If their RT exceeded 1,200 ms, they were given the feedback “even faster” (German: *noch schneller*), which appeared in red font in the middle of the screen before the next trial started. The participants did not receive any feedback on the accuracy of their response. The time course of one trial is illustrated in Fig. 1 (B: no-self-evaluation condition, C: self-evaluation condition).

Stahl et al. (2020) measured error detection and detection certainty on an 8-point rating scale ranging from “certainly right” to “certainly wrong” and found that participants tended to mainly use the extreme values of the scale. Therefore, we split the original scale into two parts and assessed error detection and response certainty separately. First, the participants evaluated whether their response was correct or erroneous by pressing the force-sensitive key with their index or middle finger of the right hand, respectively. For this evaluation, the participants were given a response time limit of 3,000 ms. Next, the participants were asked to rate their certainty of their former evaluation using the four keys assigned to their left hand. The rating ranged from certain to uncertain and was supported by a presentation of matching squares on the screen. For this rating, the participants were again given a response time limit of 3,000 ms. We did not counterbalance the responding hands for the two-staged rating procedure to avoid confusion of the participants (as reported by some participants after the experiment of Stahl et al., 2020). We ensured that the intertrial interval between the primary task and the first rating was long enough for error processing mechanisms to proceed before any possible shifts in topography due to the build-up of readiness potentials could occur and that the time window for the rating response was long enough to prevent overlaps with processes related to previous or subsequent responses. After the certainty rating, the trial was completed, and the next trial started. The intertrial interval randomly varied in length (700, 800, or 900 ms). The type and position of the symbols appearing in the white squares were randomized and distributed equally throughout each block of the experiment.

The experiment consisted of two parts. During the first part, there was neither a self-evaluation nor a certainty rating but only the main task (i.e., *no-self-evaluation condition*). We did not prolong the intertrial interval in this condition so that it matches the length of the evaluation and certainty rating as such a long black screen interval evokes mind-wandering, detachment from the task, boredom, and fatigue. These processes would limit the comparability of the two self-evaluation conditions. The aggregated cognitive load during the main task in the self-evaluation condition is probably higher than in the no-self-evaluation condition due to the anticipation that one has to evaluate one’s own performance, which is the process we wanted to manipulate. We did not implement an additional task in the no-self-evaluation condition as compensation since an additional task would set off other cognitive processes we cannot control. The no-self-evaluation condition consisted of seven blocks with a total of 394 trials (approximately 15 minutes, including breaks). The first two blocks were shorter practicing blocks with no RT limit; the other 5 blocks consisted of 64 trials each. Within a block, each symbol-position combination occurred once. The evaluation and the certainty rating were displayed in the second part of the experiment (*self-evaluation condition*). This condition consisted of 11 blocks with 64 trials each, leading to a total number of 704 trials (approximately 60 minutes, including breaks). Between blocks, the participants were given the opportunity to rest and proceed with the next block whenever they felt ready. The second part was longer than the first part, because a higher number of trials was needed to obtain a sufficient number of signaled and non-signaled responses. The order of two parts was not counterbalanced because if the self-evaluation part had preceded the no-self-evaluation condition, participants would have potentially kept evaluating their responses—even if the specific instruction had been omitted.

### Apparatus

Force sensors (FCC221-0010-L, DigiKey MSP6948-ND) that were embedded in each of the eight keys registered the response that the participants executed. The response signal of the sensors was digitized by a VarioLab AD converter at a sampling rate of 1,024 Hz. The program also captured the real-time onset of the stimuli on the computer screen via a photo sensor attached to the screen, which reacted to a change of brightness. The keypress was registered (also in real time) as a response if it exceeded a threshold of 40 cN. The response keys were adjusted to the individual length of the participant’s fingers, and they were calibrated according to the individual weight of the fingers. To prevent an excess of head movement and to ensure a constant distance to the screen (86 cm), the participant’s chin rested on a chin support during the experiment.

### Electrophysiological data

The EEG was recorded by 63 active, unipolar Ag/AgCl electrodes (Acticap, Brain Products) that were placed onto the scalp according to the standard international 10-20 system (Jasper., 1958) (FP1, FP2, AF7, AF3, AF4, AF8, F7, F5, F3, F1, Fz, F2, F4, F6, F8, FT7, FC5, FC3, FC1, FCz, FC2, FC4, FC6, FT8, T7, C5, C3, C3‘, C1, Cz, C2, C4, C4‘, C6, T8, TP7, CP5, CP3, CP1, CPz, CP2, CP4, CP6, TP8, P7, P5, P3, P1, Pz, P2, P4, P6, P8, PO7, PO3, POz, PO4, PO8, PO9, O1, Oz, O2, PO10). The electrodes were online referenced against an electrode placed on the left mastoid and they were re-referenced offline against the mean of the signal recorded at the left and right mastoid. Passive, bipolar Ag/AgCl electrodes (ExG-Amplifier, Brain Products) were used to record the electrooculogram (EOG). They were positioned horizontally next to the outer side of both eyes near the temples (horizontal EOG), as well as vertically above and below the left eye (vertical EOG). The EEG and EOG signals were continuously recorded by a BrainAmp DC amplifier (Brain Products) at a sampling rate of 500 Hz with a filter from DC to 70 Hz.

For the pre-processing of the ERP data, we locked the intervals of each trial onto the response onset with a time window ranging from 100 ms before and up to 800 ms after the response onset. We performed a baseline correction based on the 100 ms before the response onset (for MVPA 100 ms before stimulus onset). We implemented an ocular correction algorithm to correct for eye blinks in the activity patterns (Gratton et al., 1983). With an artifact rejection, we removed trials in which the ERP waves exceeded a threshold of ± 100 μV (*M* = 10.8%, *SD* = 12.2% of all trials that lay within the RT limit). We performed a current source density (CSD) analysis to detach the signal from the reference electrodes and to reduce the redundant activity of neighboring electrodes (Perrin et al., 1989). We did not apply any high-pass or low-pass filters to the data to avoid temporal smearing and amplitude reductions. The N_e/c_ amplitude was determined at the FCz electrode as the mean amplitude ±2 data points around the negative peak (i.e., we averaged the activity of five data points: the data point of the peak and two data points before and after that) in a time window of 0 ms to 150 ms after response onset. The P_e/c_ amplitude was determined at the Cz electrode as the mean amplitude ±2 data points around the positive peak and mean activity in a time window of 150 ms to 300 ms after response onset. Topographical maps (see *Results*) confirmed these electrodes as the local extrema of the two components.

### Multivariate pattern analysis

In addition to the traditional univariate analyses of ERP components at individual electrode sites, the ERP signal can also be analyzed in a multivariate fashion (i.e., at all electrode sites). Multivariate pattern analysis (MVPA) enables certain time windows to be identified, at which a person’s response can be reliably predicted from their ERP data (Bode et al., 2012). Including information from all of the electrode sites enables a more exhaustive use of information regarding the participant’s brain activity patterns, which in turn allows the identification even of small differences between the self-evaluation and the no-self-evaluation condition in the current study.

The MVPA were performed by using the support vector machine classification (SVC) from the Decision Decoding Toolbox (Bode et al., 2019). First, all of the analyses were performed for each participant separately (first-level analyses). Second, these results were averaged across all participants and inference statistics were computed (second-level analyses). We conducted a series of analyses, each contrasting two conditions: (1) no-self-evaluation condition: correct responses vs. errors; (2) self-evaluation condition: correct responses vs. errors; (3) correct responses: no-self-evaluation condition vs. self-evaluation condition; and (4) errors: no-self-evaluation condition vs. self-evaluation condition. The data were time-locked to the response onsets (i.e., the response to the symbol stimuli) with a time window ranging from 900 ms before response onset to 200 ms after response onset, which should cover the entire process from stimulus presentation to the overt response. In the first-level analysis, these time windows were subdivided into smaller nonoverlapping time windows with a length of 10 ms, each including five data points. The data points within these smaller time windows and of all of the electrodes were then transformed into vectors that formed the spatio-temporal pattern of the two conditions. A linear classifier was then trained with these vectors using the LIBSVM toolbox (Chang & Lin, 2011). The SVC process was submitted to a tenfold cross-validation, resulting in a total of 100 classification processes. The classification accuracies of these runs (i.e., the percentage of cases in which the classifier was able to sort the vectors into the conditions they belonged to) were then averaged to obtain one classification accuracy value for each of the 10-ms time windows of each participant in the respective comparison condition. For each participant, in addition to the classification accuracy, the chance classification was computed based on the empirical data. This resembled the previous procedure, except that the classification probability was obtained by permutations within a near-identical shuffled-labels analysis (Bode et al., 2019).

The second-level analyses were conducted separately for each 10-ms time window, for which the classification accuracies were averaged across all participants. The chance classification was also averaged across all of the participants. The mean classification accuracy and the chance classification were then tested against each other for every 10-ms time window within 900 ms before to 200 ms after the response onset with a series of Bonferroni-corrected *t* tests. If the mean classification accuracy deviated significantly from the chance level, then the classifier was able to decode the condition from the brain activity patterns in the respective time window.

### Statistical analyses

For the investigation of error detection in the self-evaluation condition, we conducted univariate ANOVAs with repeated measures for the factor Response Type (signaled correct, signaled error, and non-signaled error) for response rates, the percentage of multiple responses (where response force exceeded 40 cN on more than one key), RT (time span between the stimulus onset and the exceedance of 40 cN on one of the keys), response force (RF; max. force in a trial), post-response correct (percentage of trials followed by a correct response), evaluation RT (time span between the evaluation screen onset and the exceedance of 40 cN on one of the keys), response certainty, N_e/c_ and P_e/c_ mean amplitudes, and P_e/c_ mean activity. We applied Greenhouse-Geisser correction when the sphericity assumption was violated and Tukey’s HSD for within-comparisons as post-hoc tests. We compared signaled and non-signaled errors regarding error correction (percentage of errors that were immediately followed by the correct response) with a two-tailed *t*-test. The pre-post response time difference (pre-post RT_diff_) was computed by subtracting the RT following an erroneous trial from the RT preceding an erroneous trial and then calculating the mean of these differences. This procedure was described as a robust measurement for post-error slowing (Dutilh et al., 2012). For the sake of comparison, the pre-post RT_diff_ also was calculated for correct responses. We used three Bonferroni-corrected one-tailed *t*-tests to contrast pre-post RT_diff_ for signaled errors and correct responses, for non-signaled errors and correct responses, and for signaled errors and non-signaled errors. Similar to many studies (see Wessel, 2012 for discussion), we had to exclude the non-signaled correct responses from our analyses, because the majority of the participants showed less than six trials in this condition and a reliable quantification of the N_e/c_ could not be ensured (Olvet & Hajcak, 2009a). To assess the influence of self-evaluation, we compared the behavioral and neural parameters of the no-self-evaluation condition with the self-evaluation condition. For this, we performed a series of univariate ANOVAs with repeated measures for the factors Self-Evaluation (no-self-evaluation, self-evaluation) and Accuracy (correct, error) for RT, RF, pre-post RT_diff_, post-response correct, N_e/c_ and P_e/c_ mean amplitudes, and P_e/c_ mean activity.

## Results

Our first goal was to investigate error detection effects on the behavioral and neural correlates of error processing. Therefore, in a first step we report results of the self-evaluation condition, where we categorized responses into the three response types *signaled correct*, *signaled errors*, and *non-signaled errors*. Our second goal was to examine the effect of self-evaluation on participants’ performance and on error processing. Thus, in a second step we report results of the contrast between the no-self-evaluation condition and the self-evaluation condition.

### Response types

The self-evaluation condition was designed to assess neural and behavioral correlates of error processing as a variation of error detection. The descriptive statistics for all of the behavioral and neural parameters split by the three response types are depicted in Table 1.

**Table 1:** Means ± standard error of means for all assessed behavioral and electrophysiological variables separately for each Response Type (signaled correct responses, signaled errors and non-signaled errors) for n = 21 for the second part of the experiment

	Signaled correct	Signaled errors	Non-signaled errors
Response rates^a^ [%]	88.6 ± 1.8	7.4 ± 1.2	4.0 ± 0.7
Multiple responses [%]	4.9 ± 1.2	12.1 ± 1.9	44.8 ± 4.5
Response time [ms]	814.7 ± 14.1	835.2 ± 22.6	820.2 ± 31.2
Response force [cN]	162.8 ± 10.2	135.4 ± 7.6	88.3 ± 5.7
Pre-post RT_diff_ [ms]	0.1 ± 2.8	32.8 ± 12.3	85.0 ± 28.5
Post-response correct [%]	69.2 ± 3.7	69.4 ± 4.5	65.9 ± 5.8
Evaluation RT [ms]	391.5 ± 29.4	656.8 ± 50.8	489.2 ± 35.4
Certainty rating [1-4]^b^	3.7 ± 0.1	3.5 ± 0.1	2.9 ± 0.1
N_e/c_ amplitude [μV/cm^2^]	-0.16 ± 0.03	-0.26 ± 0.04	-0.23 ± 0.04
P_e/c_ amplitude [μV/cm^2^]	0.13 ± 0.03	0.33 ± 0.05	0.22 ± 0.03
P_e/c_ mean activity [μV/cm^2^]	0.11 ± 0.03	0.26 ± 0.04	0.15 ± 0.03

^a^Relative to responses from the remaining three response types. Responses which exceeded the response time limit of 1,200 ms were excluded (11.4 ± 1.7 % of all trials). Among response types, only 1.0 ± 0.3 % were non-signaled correct responses

^b^Certainty values ranged from 1 = very uncertain to 4 = very certain

#### Behavioral results

Response rates differed significantly between Response Types, *F*(1.10, 21.94) = 928.46, *p* < 0.001, ε = 0.55, η_*p*_^2^ = 0.98. From the three response types, signaled correct responses occurred more frequently than signaled and non-signaled errors, both *p* values < 0.001, whereas the two error types did not differ significantly, *p* = 0.278. The percentage of multiple responses varied significantly with Response Type, *F*(1.27, 25.40) = 80.83, *p* < 0.001, ε = 0.63, η_*p*_^2^ = 0.80. There were significantly more multiple responses for non-signaled errors compared to the other two response types, both *p* values < 0.001, whereas the signaled errors and signaled correct responses did not differ from each other, *p* = 0.094. In non-signaled error trials a correct response followed more often (43.6 ± 4.6%) than in signaled error trials (9.5 ± 1.6 %), *t*(20) = −8.97, *p* < 0.001, *d* = −1.96. The RTs did not differ significantly between Response types, *F*(1.45, 29.09) = 0.33, *p* = 0.653, ε = 0.73, η_*p*_^2^ = 0.02. The peak RF significantly varied by Response type, *F*(2, 40) = 41.59, *p* < 0.001, ε = 0.83, η_*p*_^2^ = 0.68, with signaled correct responses being the most forceful, followed by signaled errors and non-signaled errors, all *p* < 0.005. The pre-post RT_diff_ was significantly larger for signaled errors compared with correct responses, *t*(20) = −2.37, *p* = 0.042, *d* = −0.52, and larger for non-signaled errors compared to correct responses, *t*(20) = −3.02, *p* = 0.010, *d* = −0.66. The signaled errors and non-signaled errors did not differ significantly in pre-post RT_diff_, *t*(20) = −1.64, *p* = 0.348, *d* = −0.36.

The percentage of post-response correct responses did not vary significantly with Response type, *F*(2, 40) = 1.06, *p* = 0.355, ε = 0.80, η_*p*_^2^ = 0.05. The Evaluation RT varied significantly between Response types, *F*(2, 40) = 27.63, *p* < 0.001, ε = 0.85, η_*p*_^2^ = 0.58. Evaluation RT was smallest for signaled correct responses, followed by non-signaled errors and signaled errors, all *p* values < 0.026. The ANOVA for response certainty yielded a significant effect for Response type, *F*(1.23, 24.51) = 22.02, *p* < 0.001, ε = 0.61, η_*p*_^2^ = 0.52, indicating less certainty for non-signaled errors compared to signaled errors and signaled correct responses, both *p* values < 0.001. Signaled errors did not differ significantly from signaled correct responses, *p* = 0.134.

#### Electrophysiological results

**Fig. 2:**
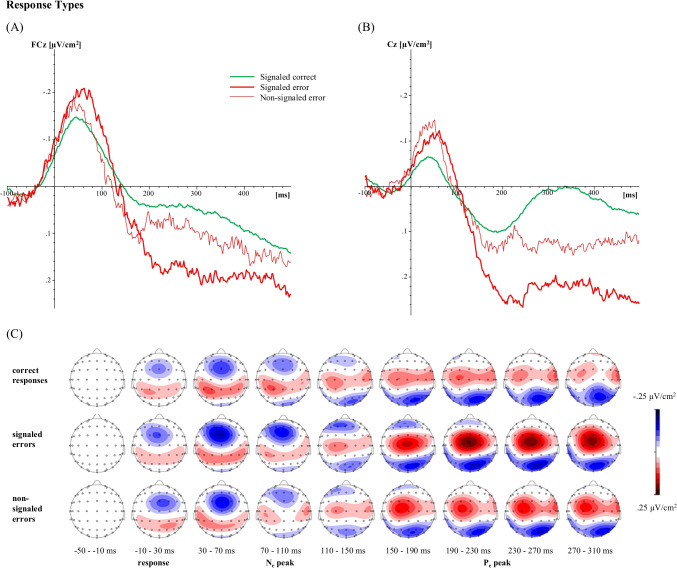
Averaged CSD-transformed waveforms of the ERP components (**a**) error negativity, measured at FCz electrode as the mean amplitude ±2 data points around the negative peak in the time window of 0-150 ms after response onset and (**b**) error positivity, measured at Cz electrode as the mean amplitude ±2 data points around the positive peak and as mean activity in the time window of 150-300 ms after response onset separately for each response type (signaled correct, signaled and non-signaled errors), as well as the respective topographical maps

The N_e/c_ mean amplitude differed significantly between Response types, *F*(2, 40) = 11.76, *p* < 0.001, η_*p*_^2^ = 0.37, with signaled errors and non-signaled errors having a larger amplitude than signaled correct responses, *p* < 0.001 and *p* = 0.005, respectively. The N_e/c_ amplitude of signaled and non-signaled errors did not differ significantly, *p* = 0.377. The P_e/c_ mean amplitude differed significantly between Response types, *F*(2, 40) = 18.11, *p* < 0.001, η_*p*_^2^ = 0.48, with signaled errors and non-signaled errors having a larger amplitude than signaled correct responses, *p* < 0.001, and *p* = 0.027, respectively. Post-hoc tests also revealed a larger amplitude for signaled errors than for non-signaled errors, *p* = 0.005. The P_e/c_ mean activity differed significantly between Response types, *F*(2, 40) = 13.02, *p* < 0.001, η_*p*_^2^ = 0.39, with signaled errors having a higher mean activity than non-signaled errors, *p* = 0.003, and signaled correct responses, *p* < 0.001. Non-signaled errors did not differ significantly from signaled correct responses, *p* = 0.360. The CSD-transformed averaged ERP waveforms together with their topographical maps are depicted in Fig. 2. We report results for the untransformed ERP data in the Supplement.

### No-self-evaluation vs. self-evaluation

In the second set of analyses, we compared the self-evaluation condition with the no-self-evaluation condition (Table 2).

**Table 2:** Means ± standard error of means for all assessed behavioral and electrophysiological variables separately for each condition (no-self-evaluation vs self-evaluation) for correct and erroneous responses for *n* = 33

	No-self-evaluation		Self-evaluation	
	Correct	Error	Correct	Error
Response rates ^a^ [%]	87.1 ± 1.3	12.9 ± 1.3	88.9 ± 1.6	11.1 ± 1.6
Response time [ms]	787.9 ± 14.0	813.4 ± 16.7	796.1 ± 12.4	804.4 ± 16.5
Response force [cN]	146.2 ± 8.2	104.6 ± 3.8	156.5 ± 8.3	107.0 ± 3.7
Pre-post RT_diff_ [ms]	-9.4 ± 2.2	52.5 ± 9.1	-0.7 ± 1.6	34.5 ± 8.9
Post-response correct [%]	77.1 ± 2.0	75.2 ± 3.1	78.3 ± 2.5	76.5 ± 2.9
N_e/c_ amplitude [μV/cm^2^]	-0.12 ± 0.02	-0.20 ± 0.03	-0.13 ± 0.02	-0.16 ± 0.02
P_e/c_ amplitude [μV/cm^2^]	0.08 ± 0.02	0.19 ± 0.03	0.07 ± 0.02	0.21 ± 0.03
P_e/c_ mean activity [μV/cm^2^]	0.02 ± 0.02	0.10 ± 0.02	0.02 ± 0.02	0.15 ± 0.02

^a^Relative to all responses within the response time limit; responses which exceeded the response time limit of 1,200 ms were excluded. The percentage of too slow responses did not differ between the two evaluation conditions (no-self-evaluation condition: 10.6% ± 1.6, self-evaluation condition: 9.9% ± 1.4, *t*(32) = 1.14, *p* = 0.264, *d* = 0.20)

#### Behavioral results

For RT, there was no significant main effect of Self-Evaluation, *F*(1, 32) = 0.00, *p* = 0.960, η_p_^2^ < 0.01, no significant main effect of Accuracy, *F*(1, 32) = 1.98, *p* = 0.169, η_*p*_^2^ = 0.06, and no significant interaction, *F*(1, 32) = 1.56, *p* = 0.221, η_*p*_^2^ = 0.05. The ANOVA for peak RF did not yield a significant main effect of Self-Evaluation, *F*(1, 32) = 1.98, *p* = 0.169, η_*p*_^2^ = 0.06, but a significant main effect for Accuracy, *F*(1, 32) = 58.13, *p* < 0.001, η_p_^2^ = 0.64, and an effect for the Self-Evaluation by Accuracy interaction, *F*(1, 32) = 5.38, *p* = 0.027, η_*p*_^2^ = 0.14. Post-hoc tests showed that correct responses were more forceful than errors in the no-self-evaluation condition, *p* < 0.001, and in the self-evaluation condition, *p* < 0.001. Correct responses did not differ significantly between self-evaluation conditions, *p* = 0.159. Errors also did not differ significantly between self-evaluation conditions, *p* = 0.961. For the pre-post RT_diff_ of errors, there was no significant difference between the no-self-evaluation and the self-evaluation condition, *t*(32) = 1.23, *p* = 0.229, *d* = 0.21. For the post-response accuracy there was no significant main effect of Self-Evaluation, *F*(1, 32) = 0.79, *p* = 0.382, η_p_^2^ = 0.02, no significant main effect of Accuracy, *F*(1, 32) = 2.14, *p* = 0.153, η_*p*_^2^ = 0.06, and no significant interaction, *F*(1, 32) = 1.01, *p* = 0.940, η_*p*_^2^ < 0.01.

#### Univariate electrophysiological results

**Fig. 3:**
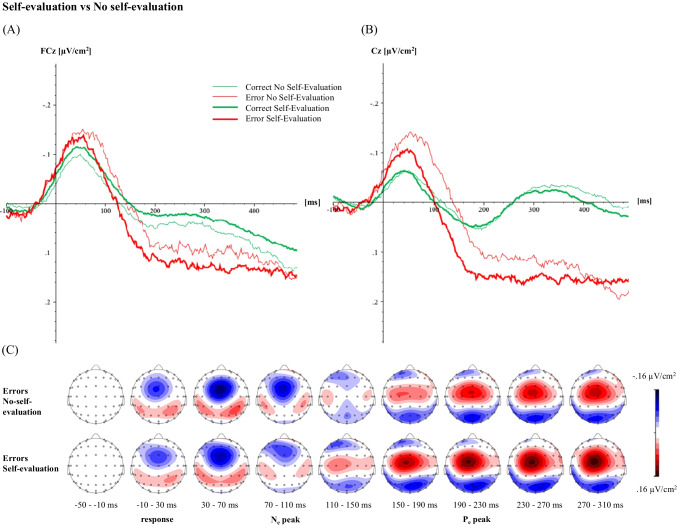
Averaged CSD-transformed waveforms of the ERP component (**a**) error negativity, measured at FCz electrode as the mean amplitude (±2 data points) around the negative peak in the time window of 0-150 ms after response onset and (**b**) error positivity, measured at Cz electrode as the mean amplitude (±2 data points) around the positive peak and as mean activity in the time window of 150-300 ms after response onset grouped by accuracy (correct, error) and experimental condition (no-self-evaluation, self-evaluation), as well as the respective topographical maps for errors of both conditions

For the N_e/c_ mean amplitude, there was no significant main effect of Self-Evaluation, *F*(1, 32) = 1.33, *p* = 0.257, η_*p*_^2^ = 0.04, but there was a significant main effect of Accuracy, *F*(1, 32) = 16.72, *p* < 0.001, η_*p*_^2^ = 0.34, with the amplitude being higher for errors than for correct responses. A significant interaction between Self-Evaluation and Accuracy, *F*(1, 32) = 5.85, *p* = 0.021, η_*p*_^2^ = 0.15, revealed a tendency for a larger N_e_ amplitude in the no-self-evaluation condition compared to the self-evaluation condition, *p* = 0.075, whereas there was no significant difference in the N_c_ between the self-evaluation conditions, *p* = 0.881. Furthermore, only in the no-self-evaluation condition was the N_e_ amplitude significantly larger than the N_c_ amplitude, *p* < 0.001, whereas they did not differ significantly in the self-evaluation condition, *p* = 0.119. For the P_e/c_ mean amplitude, there was no significant effect for Self-Evaluation, *F*(1, 32) = 0.47, *p* = 0.500, η_*p*_^2^ = 0.01, but there was an effect for Accuracy, *F*(1, 32) = 31.23, *p* < 0.001, η_*p*_^2^ = 0.49, with the amplitude being higher for errors than for correct responses. The interaction between Self-Evaluation and Accuracy was not significant, *F*(1, 32) = 1.65, *p* = 0.208, η_*p*_^2^ = 0.05. For the P_e/c_ mean activity, there was no significant effect for Self-Evaluation, *F*(1, 32) = 3.68, *p* = 0.064, η_*p*_^2^ = 0.10, but there was an effect for Accuracy, *F*(1, 32) = 26.70, *p* < 0.001, η_*p*_^2^ = 0.45. The interaction between Self-Evaluation and Accuracy was significant, *F*(1, 32) = 6.27, *p* = 0.018, η_*p*_^2^ = 0.16. Post-hoc tests revealed a higher mean activity for errors than for correct responses in the no-self-evaluation condition, *p* = 0.007, and in the self-evaluation condition, *p* < 0.001. The P_e/c_ mean activity also was higher for errors in the self-evaluation condition than in the no-self-evaluation condition, *p* = 0.017, whereas correct responses did not differ significantly between conditions, *p* > 0.999. The CSD-transformed averaged ERP waveforms together with their topographical maps are depicted in Fig. 3. We report results for the untransformed ERP data in the Supplement.

#### Multivariate electrophysiological results

**Fig. 4:**
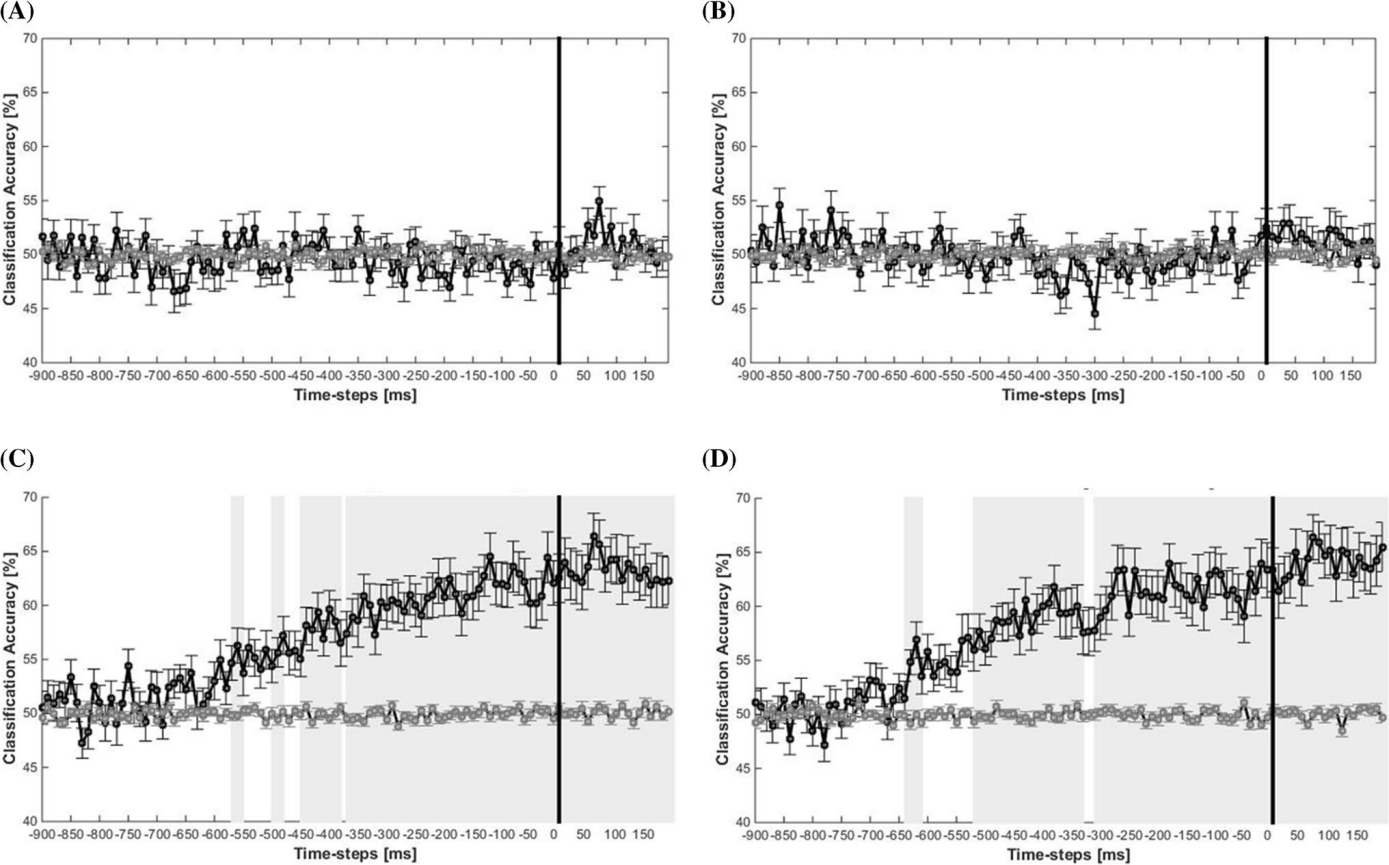
Response-locked MVPA results. Classification accuracies in percent for each time step for comparisons between correct responses and errors in the no-self-evaluation condition (**a**) and the self-evaluation condition (**b**), as well as for comparisons between evaluation conditions for correct responses (**c**) and errors (**d**). Grey areas indicate time windows in which the classification accuracies (black lines) differ significantly from the empirical chance level (grey lines). Corrected for multiple comparisons

Comparing errors and correct responses within the no-self-evaluation condition (Fig. 4a) and within the self-evaluation condition (Fig. 4b) did not reveal any time windows at which the classifier was able to significantly predict the response accuracy (errors vs. correct) from the brain activity patterns (for all 10-ms time windows, the predictions of the response accuracy were around chance level). However, the algorithm was successful in predicting the evaluation conditions (self-evaluation vs. no-self-evaluation) from brain activity within correct trials (Fig. 4c) and within error trials (Fig. 4d) significantly above chance-level. Interestingly, the classifier was able to predict the evaluation conditions already starting at 550 ms and 650 ms before response onset (within correct trials and within error trials, respectively). The respective feature weights analyses are displayed in the Supplement. In sum, this machine-learning based approach, which identifies time windows where condition-specific brain activity is decodable from the spatio-temporal activity patterns (i.e., ERP data points from all electrodes within 10-ms time windows), showed that the self-evaluation and the no-self-evaluation condition differ in these patterns at a very early stage of processing and during the entire period even after the response.

The additionally performed MVPA including RT-matched comparisons, accounting for differences in RTs between conditions, reflected similar patterns with significant time windows starting 500 ms before response onset for correct responses and 600 ms before response onset for errors (which is not shown for the sake of brevity).

### Subsequent exploratory analyses

**Fig. 5:**
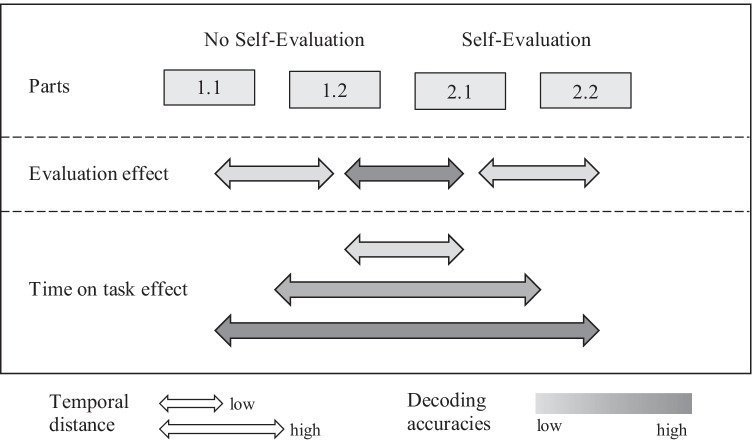
Assumptions regarding the classification accuracies of the contrasted parts based on the split-logic of both conditions. Evaluation effect: Classification accuracies are higher for cross-condition comparisons compared to within-condition comparisons of the same temporal distance. Time on task effect: Classification accuracies are higher for contrasts between parts with large temporal distance compared to parts with small temporal distance

The remarkable differences in brain activity patterns several hundred milliseconds before response onset between the self-evaluation conditions, irrespective of the response type, could indicate a broader influence of self-evaluation on brain activity patterns preceding a response. However, due to the constant order of both conditions, we cannot rule out the possibility that the differences reflect changes in more general processing mechanisms over the course of the experiment (e.g., training, attention, vigilance). To further assess whether the MVPA results reflect an effect of self-evaluation, an effect of time on task or a combination of both, we carried out several subsequent exploratory analyses. We made a within-condition split, resulting in four parts (1.1, 1.2, 2.1 and 2.2), which we then contrasted against each other using MVPA (for an overview, see Fig. 5). If the previous MVPA results are based on a time on task effect, then in our subsequent MVPA the classification accuracies for contrasts between two parts should increase with temporal distance between the two contrasted parts (i.e., part 1.1 vs. part 2.2 should show the highest classification accuracies). If the MVPA effects were due to self-evaluation, then the classification accuracies should be higher for contrasts between cross-conditional parts compared to contrasts between within-conditional parts, which have the same temporal distance. In other words, accuracies should be higher for contrasting part 1.2 with part 2.1 than for contrasting the parts within the no-self-evaluation condition (1.1 vs. 1.2) and contrasting the parts within the self-evaluation condition (2.1. vs. 2.2). Accordingly, when we find increased decoding accuracies for temporally more distant parts and an increased decoding accuracy for the cross-conditional parts, then this would suggest and influence of both time on task and self-evaluation.

Because the differences in brain activity patterns were not error specific, we collapsed errors and correct responses for the subsequent exploratory analyses. This prevents an exclusion of a substantial number of datasets due to an insufficient number of error trials. It also enables the inclusion of the 33 datasets that we analyzed previously. We also analyzed behavioral measures for the four parts. We conducted additional analyses of behavioral data in a blockwise manner (5 blocks for the no-self-evaluation condition and 11 blocks for the self-evaluation condition) to test for time on task effects in the participants’ behavior. We analyzed RT and RF collapsed over response types. To capture time on task effects on the level of response rates, we also analyzed the error rates of the different parts and blocks.

#### Behavioral results

A one-way ANOVA with the factor part (1.1, 1.2, 2.1, 2.2) showed no significant variation for error rate: *F*(1.95, 62.40) = 1.87, *p* = 0.163, ε = 0.65, η_*p*_^2^ = 0.06. RT varied significantly by part: *F*(1.83, 58.65) = 4.92, *p* = 0.013, ε = 0.61, η_*p*_^2^ = 0.13. Post-hoc tests revealed that RT was slower in part 1.1 (804.9 ± 13.2 ms) than in part 1.2 (779.6 ± 14.4 ms), *p* = 0.003, and slower in part 1.2 compared with part 2.2 (799.0 ± 12.3 ms), *p* = 0.032. The other parts did not differ significantly: all *p* values > 0.201. The peak RF did not differ significantly between parts: *F*(1.60, 51.17) = 2.06, *p* = 0.147, ε = 0.53, η_*p*_^2^ = 0.06. Comparing the blocks within the two self-evaluation conditions, neither the no-self-evaluation condition, *F*(3.15, 100.88) = 1.77, *p* = 0.156, ε = 0.79, η_*p*_^2^ = 0.05, nor the self-evaluation condition, *F*(5.45, 141.75) = 0.98, *p* = 0.439, ε = 0.55, η_*p*_^2^ = 0.04, showed a significant variation in error rates with block. The ANOVA for RT in the no-self-evaluation condition yielded a significant effect of block: *F*(4, 128) = 6.15, *p* < 0.001, ε = 0.83, η_*p*_^2^ = 0.16. Post-hoc tests revealed that RTs were slower for the first block compared with the last two blocks, both *p* values < 0.005, and RTs in the second and the third block were both slower than in the last block, both *p* values < 0.032. The other blocks did not differ significantly, all *p* values > 0.071. In the self-evaluation condition, RT did not differ significantly between blocks: *F*(6.39, 204.35) = 0.86, *p* = 0.530, ε = 0.64, η_*p*_^2^ = 0.03. In the no-self-evaluation condition, there was a significant effect of block on peak RF: *F*(2.82, 90.26) = 3.78, *p* = 0.015, ε = 0.71, η_*p*_^2^ = 0.11. Post-hoc tests revealed that responses were more forceful in the first block compared with the last two blocks: both *p* values < 0.028. The other blocks did not differ significantly in peak RF: all *p* values > 0.175. In the self-evaluation condition, for the peak RF there was no significant effect of block:, *F*(4.16, 133.12) = 1.43, *p* = 0.225, η_*p*_^2^ = 0.04.

#### MVPA results

**Fig. 6:**
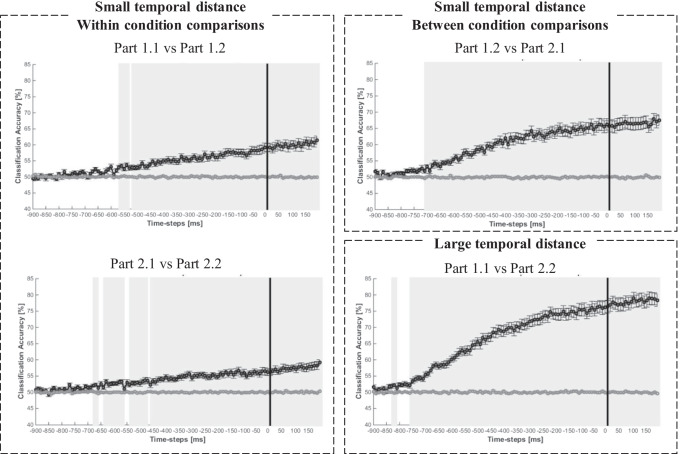
Classification accuracies in percent for each time step for comparisons between the parts with small temporal distance within conditions (part 1.1 vs. part 1.2 and part 2.1 vs. part 2.2), with small temporal distance between conditions (part 1.2 vs. part 2.1) and with the largest temporal distance (part 1.1 vs. part 2.2). Grey areas indicate time windows in which the classification accuracies (black lines) differ significantly from the empirical chance level (grey lines)

The MVPA results are depicted in Fig. 6. To test whether the classification accuracies are significantly higher for certain decoding groups (i.e., contrasts between two parts) compared with others, we computed aggregated classification accuracy scores for each individual, decoding group, and condition. To this end, we subtracted the empirical chance accuracy from the classification accuracy for each time step of each decoding group for each participant. The resulting difference scores indicate the percentage of additional trials that the classifier successfully decoded correctly when given the nonshuffled compared with the shuffled data. Finally, we averaged these scores across time steps. For every participant, each decoding group is now represented by a single difference value that represents its mean classification accuracy under consideration of chance accuracy. With these difference scores, we conducted a one-way ANOVA with repeated measures for the factor Decoding Group to test whether the classifier achieved higher classification accuracies for certain decoding groups compared with others.

The ANOVA showed that the aggregated accuracy scores indeed varied significantly between decoding groups: *F*(2.87, 91.72) = 96.11, *p* < 0.001, ε = 0.57, η_*p*_^2^ = 0.75. Post-hoc tests revealed that the accuracy scores were higher for the cross-condition decoding group with small temporal distance (part 1.2 vs. 2.1, *M* = 10.2 %, *SE* = 1.1%; i.e., the classifier sorted on average an additional 10.2% of the trials into the correct condition compared to chance classification) compared with both within-condition decoding groups with small temporal distances (part 1.1 vs. 1.2, 5.0 ± 0.6%; and part 2.1 vs. part 2.2, 4.2 ± 0.5%), both *p*s < 0.001, whereas the within-condition decoding groups did not differ, *p* = 0.907. Post-hoc tests also revealed that the scores were highest for the decoding group contrasting the temporally most distant parts (part 1.1 vs. part 2.2, 17.4 ± 1.2%), followed by the decoding groups contrasting parts of medium temporal distance (part 1.1 vs. 2.1, 14.4 ± 1.2% and part 1.2 vs. part 2.2, 14.1 ± 1.1) and then the decoding groups comparing parts of the smallest temporal distances: all *p* values < 0.003. The two decoding groups that compare parts of medium temporal distance did not differ, *p* = 0.999. The respective feature weights analyses are displayed in the Supplement.

## Discussion

Using the recently developed 8ART (Stahl et al., 2020), we examined the neural and behavioral features of error processing in a complex task with challenging response selection. In the first step, we assessed variations in neural and behavioral correlates of error processing with error detection in this novel task. The second step investigated the influence of self-evaluation in terms of response accuracy on error processing by extending traditional univariate ERP analyses with the more modern MVPA approach. Finally, we explored how the behavioral parameters and MVPA results varied with time on task.

### Error detection and error processing in the 8ART

In our modified version of the 8ART, we were able to uncover variations in error processing mechanisms with error detection. This supports the previous findings in the literature from more simple task designs. In contrast to Stahl et al. (2020), we found the common N_e/c_ effect (Nieuwenhuis et al., 2001) of response type in our study irrespective of response speed, whereas Stahl et al. (2020) only found a N_e/c_ difference for fast trials. Due to the structure of the present study, we suppose that the representation of the stimulus-response mapping was much more trained by the first part, which comprised more than 300 trials (Stahl et al. only used 64 practice trials). Therefore, a better-established memory representation might have enabled higher sensitivity of the N_e/c_ related error process. The P_e/c_ effect was replicated in terms of an increased P_e_ amplitude for signaled errors compared to non-signaled errors and correct responses. This supports the notion that error evidence accumulation proceeds similarly for binary (Steinhauser & Yeung, 2010; Steinhauser & Yeung, 2012) and more complex choice tasks (Stahl et al., 2020). The behavioral results fit the findings of Stahl et al. (2020), which indicates that our task modifications did not affect the previously reported patterns.

Interestingly, the non-signaled errors in our task stand out from the common literature findings regarding P_e_ amplitude, evaluation RTs, and pre-post RT_diff_ (Endrass et al., 2007, 2012; Klein et al., 2007; Nieuwenhuis et al., 2001; Wessel et al., 2011), which were all larger than for correct responses. The high number of multiple responses and response corrections in non-signaled errors appear to be responsible. These additional responses might have induced uncertainty about response accuracy (e.g., when participants pressed the correct key immediately after an incorrect key) and impeded error evidence accumulation reflected by a smaller but not missing P_e_ amplitude, and (ultimately) prevented successful error signaling. Indeed, non-signaled errors were associated with the lowest response certainty. In our complex paradigm, error evidence accumulation is probably time consuming particularly for non-signaled errors due to the high number of multiple responses. This was reflected in the evaluation RTs (signaled errors > non-signaled errors > correct responses), which suggests that the time spent on response evaluation is an indicator for the amount of error evidence that is accumulated over time. Thus, the gradual variation of the P_e/c_ (reflected by P_e/c_ amplitude) might be more realistic compared with an all-or-nothing principle (reflected by P_e/c_ mean activity). Endrass et al. (2012) also found evidence for the P_e/c_ being a gradual measure of error awareness. Yet, an enhancement only for signaled errors is commonly reported in literature (Nieuwenhuis et al., 2001), which underlines the impact of different ERP quantifications and the importance to discuss deviations between them.

Finally, non-signaled errors feature a large pre-post RT_diff_, i.e., post-error slowing, which might have served as a mechanism to reduce the uncertainty associated with non-signaled errors in the next trial. This contrasts previous findings of increased post-error slowing for signaled but not for non-signaled errors (Endrass et al., 2007, 2012; Klein et al., 2007), even when more response corrections for non-signaled errors were observed (Endrass et al., 2007). In our study, the consecutive evaluation process (detection and certainty ratings) might have given participants the opportunity to process their response more deeply and to continue error processing after an initial detection response. Therefore, although error evidence might not have been sufficient by the time the response was inaccurately signaled as correct, subsequent error processing might have led to error detection and therefore increased post-error slowing in the next trial. Post-error slowing then reflected the prolongation due to orienting and adaptational mechanisms elicited not only by the previous incorrect trial but also by the previous incorrect response signaling, which would support a relation of post-error slowing to the perceived correctness of a response (Endrass et al., 2012).

### Self-evaluation and error processing

Our second goal was to investigate whether self-evaluation influences error processing and behavioral performance. Comparing the self-evaluation and the no-self-evaluation condition did not reveal differences regarding behavioral measures. Assuming that self-evaluation increases error significance, this is in line with the finding that the effects of error significance do not always translate to the participants’ performance (Ganushchak & Schiller, 2008; Hajcak et al., 2005; Maruo et al., 2016). Similarly, Grützmann et al. (2014) did not find significant differences in error rates and post-response accuracy improvement between the two self-evaluation conditions. However, while post-error slowing was showing in the no-self-evaluation condition, the researchers reported slower RTs in general and no post-error slowing in the self-evaluation condition. They concluded that a more cautious response mode in the self-evaluation condition led to a ceiling effect in RTs that hindered post-error slowing. In our study, the RTs in the two evaluation conditions were similar and therefore the absence of RT ceiling effects may explain the observed post-error slowing in both the self-evaluation and the no-self-evaluation condition. Interestingly, the pattern of evaluation RTs we found in our study (signaled error > non-signaled error > signaled correct) fits the interpretation of Grützmann et al. (2014), who also observed slower evaluation RTs for errors than for correct responses. This pattern indicates that post-error slowing already occurs during the self-evaluation process and reflects an orienting response to expectancy violating events (Grützmann et al., 2014; Wessel, 2018).

Surprisingly, we found an N_e/c_ difference only in the no-self-evaluation condition and a tendency for a reduced N_e_ in the self-evaluation condition. In case of a clear effect of self-evaluation induced by an enhanced error significance or attention to error monitoring, error processing should have been reinforced in the self-evaluation condition. The inversed effect, which is opposed to what Grützmann et al. (2014) found, can have various reasons. First, the literature has shown that error monitoring is modulated by task difficulty (Kaczkurkin, 2013; Riesel et al., 2015; Schreiber et al., 2012; Somon et al., 2019; Voodla & Uusberg, 2021) and uncertainty (Bultena et al., 2020; Pailing & Segalowitz, 2004; Scheffers & Coles, 2000; Selimbeyoglu et al., 2012). While the N_e_ amplitude is found to be attenuated, the N_c_ amplitude increases with task difficulty and uncertainty, presumably due to a hindered error detection process (Pailing & Segalowitz, 2004). Our results showed that the difference between the N_e_ and N_c_ in the self-evaluation condition was significantly smaller than in the no-self-evaluation condition and that the N_e_ tended to be smaller in the self-evaluation condition than in the no-self-evaluation condition. These findings suggest that an effect of task difficulty or uncertainty might have counteracted an effect of error significance. However, studies that reported N_e/c_ modulations also reported modulations of behavioral parameters by task difficulty (Pailing & Segalowitz, 2004; Riesel et al., 2015; Schreiber et al., 2012; Somon et al., 2019; Voodla & Uusberg, 2021). An effect of task difficulty was not reflected in our behavioral data, because the two self-evaluation conditions did neither differ significantly regarding error rates, RTs, post-response slowing, and post-response accuracy improvement nor regarding the percentage of too slow responses, which speaks against an increased task difficulty in the self-evaluation condition. Moreover, our participants reported an overall high certainty in their responses, and there was no enhancement of N_c_ amplitude, which also argues against a higher task difficulty in the self-evaluation condition.

Second, we cannot preclude that the decrease in N_e_ amplitude might indicate an effect of time on task (Boksem et al., 2006; Horváth et al., 2020; Tops et al., 2006; Tops & Boksem, 2010). However, the part- and blockwise analyses of the behavioral data revealed neither systematic increases in error rates nor systematic variations in RT (RT increase as an indicator of fatigue; continuous RT decrease as an indicator of practice), which we would expect in case of a relevant time on task effect (Horváth et al., 2020; Tops & Boksem, 2010). In addition to that, it has been shown that increasing the motivation to engage with the task, e.g., by implementing external monetary incentives, counteracts a time on task induced N_e_ decrease (Tops & Boksem, 2010). We assume that our self-evaluation rating increased participants’ motivation to direct more attention to the task to prevent a negative self-evaluation, reducing the likelihood of a mere time on task effect as the single cause for a decrease in N_e_ amplitude and the absence of a significant N_e/c_ difference in the self-evaluation condition.

Third, individual differences also can account for the absence of an error significance effect. Previously, it has been shown that for some individuals errors have a higher significance and are processed more deeply (Riesel et al., 2012). For example, punishment substantially modified error monitoring in highly anxious participants (Riesel et al., 2012, 2019). In this notion, it is possible that in our more complex task, an effect of error significance might only unfold in individuals that display an increased sensitivity to modulations of error significance. Hence, including traits such as anxiety (Riesel et al., 2012), perfectionism (Stahl et al., 2015), or narcissism (Mück et al., 2020) as moderators in future studies will help to further elucidate the relationship between self-evaluation and error processing in our more complex task.

Last and most importantly, we used two scales: the binary detection scale and the four-point certainty evaluation. The consecutive and thus prolonged self-evaluation process in our task might have absorbed more attentional resources than the single three-level rating scale Grützmann et al. (2014) used in their study. This might have led to less cognitive resources available for error monitoring in the primary task and therefore to a decreased N_e_ amplitude in the self-evaluation condition. Grützmann et al. (2014) argued that, although they found an increased N_e_ amplitude in their self-evaluation condition indicating an enhanced error significance, it is still possible that this effect was weakened by the absorption of attentional resources through the rating procedure, but the influence of error significance was more impactful. In our task where the ratings were more complex, it might in fact have been the other way around.

We did not find a variation of P_e/c_ amplitude with self-evaluation. However, P_e/c_ mean activity showed a clear effect of self-evaluation, with a higher mean activity for errors in the self-evaluation condition compared with errors in the no-self-evaluation condition. This indicates that self-evaluation increases the need for processing errors more deeply and the motivation to intensify the evidence accumulation process. While early error processing mechanisms reflected by the N_e/c_ might have been reduced because the subsequent rating procedure absorbed attentional resources, error evidence accumulation reflected by the P_e/c_ might be exactly the process that absorbs the attentional resources. We assume that error evidence accumulation is the relevant process to successfully detect the response accuracy. Therefore, more attentional resources might have been directed towards error evidence accumulation, while they were withdrawn from early, fast error monitoring.

### Self-evaluation and whole-brain activity

Interestingly, the more sensitive classifier was unable to identify a difference in whole-brain activity pattern between errors and correct responses. Instead, it identified differences between the two self-evaluation conditions before and after the response. The difference between the findings of Bode and Stahl (2014), who reported significant error-related differences in a two-alternative task, might be related to the more complex nature of our task. Due to the high number of response alternatives and multiple responses, brain activity patterns of correct responses and errors might have been more similar to each other and intermixed with other processes. The complex response selection and the more complex stimulus-response assignment might have induced more “noise” and thus challenged the decoding of an unequivocal error-related pattern. Surprisingly, the self-evaluation conditions seemed to differ already on early processing stages (more than 500 ms before response onset). Because we already precluded error-specific differences as a cause for this effect, we conducted additional MVPA to investigate the factors that contributed to the remarkable differences. Subsequent analyses revealed that the large differences between the two evaluation conditions were in parts due to time on task, because the classifier was more successful in identifying the different experimental parts when the temporal distance between them was larger. Fluctuations in processes, such as fatigue, vigilance, and attention, might be the underlying sources for this effect. Moreover, self-evaluation seemed to amplify this established time on task effect, because the classifier also was more successful in differentiating self-evaluation from no-self-evaluation parts than parts from the same condition. One possible explanation for this might be that the two self-evaluation conditions differ in the allocation of attentional resources. While the primary task was the same for both conditions, there might have been less attentional resources available for the primary task in the self-evaluation condition due to the complex rating procedure. This might explain the early differences between the two evaluation conditions and is in line with our ERP results. We can preclude the influence of confounds such as error rates, RT, and RF on our MVPA results, because these did not vary systematically with the observed MVPA patterns.

### Limitations

Despite our successful detection of behavioral and neural differences dependent on response type, as well as our interesting findings regarding self-evaluation, our study is marked by some limitations. First, the second part of the experiment had more trials than the first part, which enables a sufficient number of signaled and non-signaled error trials but limits comparability. We decided against prolonging the first part to avoid fatiguing the participants. To ensure that the measurement of the N_e/c_ is equally stable in both conditions, we conducted trial-matched ERP analyses, which we report in the Supplement.

Second, unfortunately yet unsurprisingly (see Wessel, 2012 for a discussion), our participants did not produce a sufficient number of non-signaled correct responses, which led us to exclude this response type from analyses.

Third, we had to exclude a considerable number of participants from the analyses, because they did not produce enough non-signaled errors, whereas a larger sample size would have been preferable. We further inspected the data sets to ensure the effectiveness of our task design and data quality for the first set of analyses. In the past, a low number of non-signaled errors has been a common issue in error awareness studies (Kirschner et al., 2021) that often led to the exclusion of a substantial number of participants (Niessen et al., 2020; O’Connell et al., 2007; Scheffers & Coles, 2000). In a recent study, we obtained a reasonable rate of non-signaled errors with our newly developed 8ART (Stahl et al., 2020; 5.6 ± 0.7% non-signaled errors, 5 of 38 participants excluded due to an insufficient number of non-signaled errors) and thus showed that the task is well-suited to investigate error awareness. To preclude systematic differences between the data sets included in our study and those excluded due to an insufficient rate of non-signaled errors, we compared both subsamples. The subsamples did not differ significantly in behavioral parameters regarding RT, peak RF, or post-response slowing (for statistics see Supplement). Instead, the first part of our study probably led to a more mature error-signaling process in the second part, which might be responsible for the rate of low non-signaled errors. Therefore, either further increasing task complexity to foster non-signaled errors or carefully reducing the opportunity for practice will be an important step in our future studies.

Fourth, the blockwise analyses show a change in performance that is stronger in the beginning of the experiment. This shift in performance is limited to RT, whereas the error rates are stable across the experiment. This nonlinear change in performance most likely reflects an effect of practice. This might be a problem for the partwise MVPA that imply a more linear change of neural activity with time on task. To ensure that the partwise MVPA are not distorted by the nonlinear change in RT, we conducted RT-matched partwise MVPA. The results are reported in the Supplement. The analyses show that the nonlinear change in RT does not distort the partwise MVPA effects.

Moreover, in our analyses we did not exclude trials that followed timeout feedback (i.e., where the participant responded too slow in the previous trial). The timeout feedback might temporarily shift the focus to speed rather than accuracy so that subsequent errors might occur because the participant tries to meet the response time limit. However, we consider it as a benefit that our task captures a variety of error types, e.g., errors due to speed pressure (which can occur with or without previous timeout feedback), errors due to a failed response selection process, or premature responses. This relates more closely to errors committed in everyday life. In the same note, we also did not exclude trials where multiple responses were executed (which were most prevalent in non-signaled errors). The execution of multiple responses might influence error processing mechanisms, because it impedes a clear identification of the response that was performed first, thus hindering the assessment of whether or not this response deviates from the correct response. However, on a conceptual level it is difficult to separate the execution of multiple responses from non-signaled errors, as a great portion of errors might only be non-signaled because of the multiple responses. Thus, excluding trials with multiple responses would limit the representativeness of our results, as we would ignore an important cause for non-signaled errors.

Finally, we opted for a constant order where the no-self-evaluation condition was always presented first. This results in a possible confound between the time on task and an effect of self-evaluation. We deliberately restrained from counterbalancing the two self-evaluation conditions, because we suspected an interaction between time on task and self-evaluation, in which case counterbalancing is not helpful to disentangle time on task from other effects (Hutchison et al., 1999; Keppel, 1982; Sayette et al., 2010). A carryover effect from the self-evaluation condition to the no-self-evaluation condition appeared highly plausible. If explicitly instructing participants to evaluate their response accuracy enhances the attentional resources allocated to this process (Grützmann et al., 2014), and if we had instructed participants to do so in the first part of the experiment, it is likely that this enhancement would have transferred to the second part of the experiment, even if the rating was not present. This is problematic, because we do not expect such a carryover effect when the no-self-evaluation condition precedes. Therefore, counterbalancing would interact with the order of conditions. Many researchers have precluded or at least warned about the implementation of counterbalancing whenever differential carryover effects are presumed, because they impede the interpretability of results (Keppel, 1982; Sayette et al., 2010; Winer, 1971). Previous studies that implemented counterbalancing or trial-by-trial variations risked capturing these differential carryover effects which stayed unanalyzed in most of the studies. Our design precludes differential carryover effects, albeit at costs of possible time on task effects. By implementing several MVPA, we were still able to uncover an effect of self-evaluation that exceeds a time on task effect on brain activity patterns. We discuss the effects of time on task and the issue of counterbalancing more deeply in the Supplement.

### Conclusions

In this study, we have implemented the recently developed 8ART. This has enabled us to demonstrate that in a more complex task with higher cognitive load, neural mechanisms of error processing vary with error detection. This is similar to the findings in the literature that used binary response tasks. We successfully replicated Stahl et al.’s (2020) findings regarding behavioral parameters and the later stages of error processing. We also observed the common N_e/c_ effect (in contrast to Stahl et al., 2020), presumably due to the extended learning phase in our study. These results encourage further studies to implement more complex tasks that more accurately reflect decision-making processes in everyday life. We did not observe that self-evaluation enhanced early error processing mechanisms by increasing error significance. Instead, the absorption of attentional resources by the rating procedure might be responsible for the decrease in early error processing mechanisms we observed. Self-evaluation enhanced later error processing mechanisms, which presumably reflect error evidence accumulation. While early error processing might have been decreased due to the withdrawal of attentional resources, we assume that error evidence accumulation was exactly the process that absorbed the attentional resources, because it is crucial to successfully detect response accuracy. Beyond the influence of self-evaluation, we uncovered changes in earlier and broader processing mechanisms developing with time on task. These findings support the notion that a more holistic approach that exhausts information to a larger extent can complement traditional univariate approaches by further disentangling variations in brain activity patterns. Together, the different methodological approaches deepened our insight into the relation of self-evaluation and error processing in our complex task.

## Supplementary information

ESM 1 (PDF 14 mb)
