# Supplementary material for: The influence of error detection and error significance on neural and behavioral correlates of error processing in a complex choice task
Source: Cogn Affect Behav Neurosci. 2022 Aug 2;22(6):1231–49. doi: 10.3758/s13415-022-01028-6 (PMC9622536; doi:10.3758/s13415-022-01028-6)
Supplement: Supplementary file 1 — (PDF 14 mb) [file 13415_2022_1028_MOESM1_ESM.pdf]

The influence of error detection and error significance on neural and behavioral correlates of error processing in a complex choice task

Elisa Porth, André Mattes, Jutta Stahl

Supplementary materials

Supplementary materials

Comparison of excluded and included subsamples

For RT, there was no significant effect of Sample, $F(1, 36) = 1.41$, $p = 0.244$, $\eta_p^2 = .04$, and there was no significant Response Type-by-Sample interaction, $F(1.21, 43.47) = 0.95$, $p = 0.351$, $\eta_p^2 = .03$. For peak RF, there was no significant effect for Sample, $F(1, 36) = 2.09$, $p = 0.157$, $\eta_p^2 = .05$, and no significant Response Type-by-Sample interaction, $F(1.40, 50.47) = 0.95$, $p = 0.364$, $\eta_p^2 = .03$. For the pre-post RT_{diff}, neither Sample, $F(1, 35) = 1.65$, $p = 0.207$, $\eta_p^2 = .05$, nor the interaction of Response Type and Sample, $F(1.21, 42.38) = 0.39$, $p = 0.578$, $\eta_p^2 = .01$, yielded significant effects.

Electrophysiological results of the untransformed event-related potentials

The descriptive statistics of the untransformed ERP data of the three response types are depicted in Table 1.

Table 1.
Means ± standard error of the untransformed data for the $N_{e/c}$ amplitude, the $P_{e/c}$ amplitude and the $P_{e/c}$ activity separately for each Response Type (signaled correct responses, signaled errors and non-signaled errors) for n = 21.

	**Signaled correct**	**Signaled errors**	**Non-signaled errors**
$N_{e/c}$ amplitude [µV]	-2.1 ± 0.5	-5.8 ± 0.8	-5.3 ± 0.7
$P_{e/c}$ amplitude [µV]	0.6 ± 0.7	5.6 ± 1.0	4.3 ± 0.9
$P_{e/c}$ mean activity [µV]	0.3 ± 0.6	4.0 ± 1.1	2.1 ± 0.8

The pattern of results for the $N_{e/c}$ amplitude was similar to the CSD transformed data. The $N_{e/c}$ amplitude varied significantly with response types, $F(1.39, 27.79) = 11.26$, $p < 0.001$, $\eta_p^2 = .36$. The $N_{e/c}$ amplitude was larger for signaled errors and non-signaled errors compared to signaled correct responses, $p < 0.001$ and $p = 0.002$, respectively, and the two error types did not differ significantly, $p = 0.799$. For the $P_{e/c}$ the pattern of results slightly differed from the CSD analyses. The $P_{e/c}$ amplitude also varied with Response Type, $F(2, 40) = 12.93$, $p < 0.001$, $\eta_p^2 = .39$, and was larger for signaled and non-signaled errors compared to signaled correct responses, $p < 0.001$ and $p = 0.002$, respectively. Opposed to the CSD transformed data, the two error types did not differ significantly in $P_{e/c}$ amplitude, $p = 0.415$. Analogously to the CSD transformed data, the $P_{e/c}$ mean activity varied significantly with response type, $F(2, 40) = 6.67$, $p = 0.003$, $\eta_p^2 = .25$, and was larger for signaled errors compared to

signaled correct responses, $p = 0.002$, while it did not differ significantly between non-signaled errors and correct responses, $p = 0.197$. The difference between signaled and non-signaled errors was not significant, $p = 0.154$. The untransformed grand average waveforms are depicted in Figure 1.

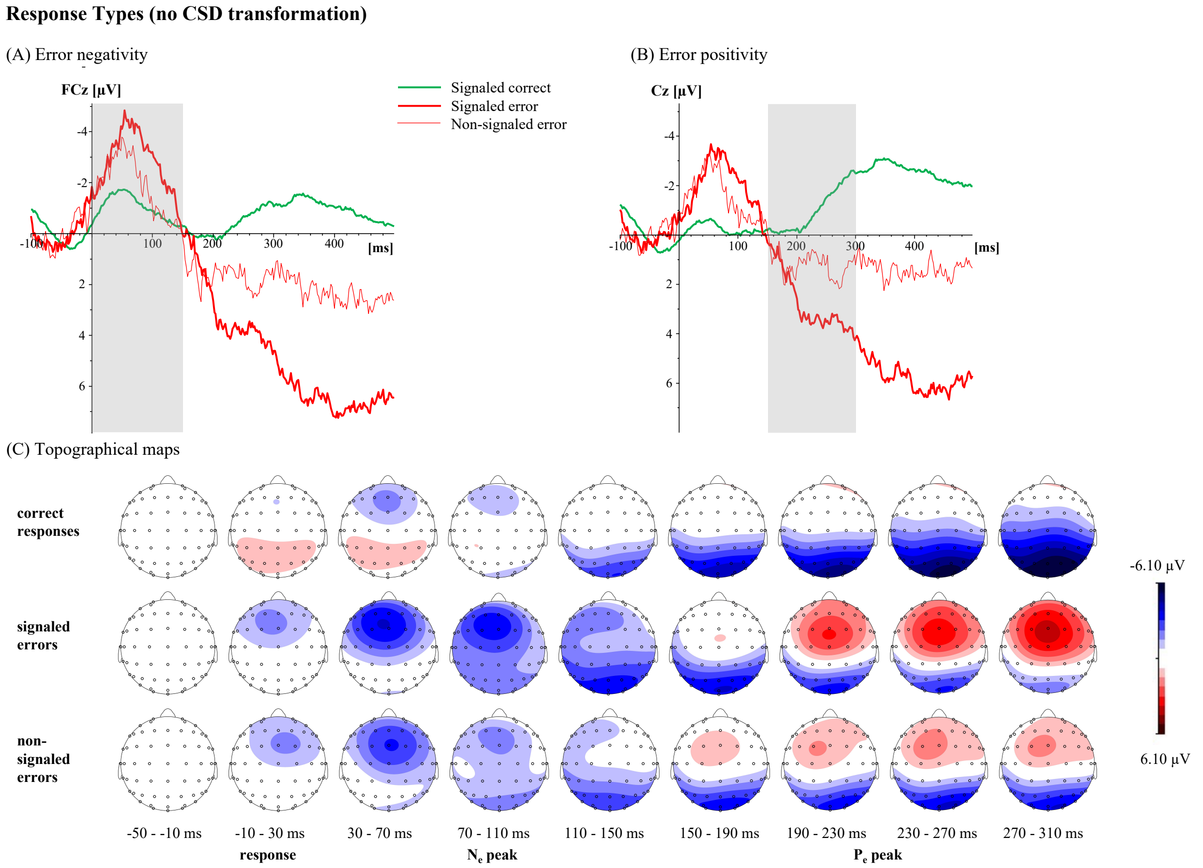

Figure 1. Averaged untransformed waveforms of the ERP component (A) error negativity, measured at FCz electrode as the mean amplitude (± 2 data points) around the negative peak in the time window of 0-150 ms after response onset and (B) error positivity, measured at Cz electrode as the mean amplitude (± 2 data points) around the positive peak and as mean activity in the time window of 150-300 ms after response onset grouped by response type, as well as the respective topographical maps for errors of both conditions.

The descriptive statistics of the untransformed ERP data from the no-self-evaluation condition and the self-evaluation condition are depicted in Table 2.

Table 2

Means ± standard error of means of the untransformed data for the $N_{e/c}$ amplitude, the $P_{e/c}$ amplitude and the $P_{e/c}$ activity for correct and erroneous responses of the no-self-evaluation and the self-evaluation condition.

	**No-self-evaluation**		**Self-evaluation**	
	**Correct**	**Error**	**Correct**	**Error**
$N_{e/c}$ Amplitude [µV]	-2.3 ± 0.4	-5.7 ± 0.5	-2.4 ± 0.4	-4.4 ± 0.4
$P_{e/c}$ Amplitude [µV]	-0.3 ± 0.5	3.5 ± 0.7	-0.4 ± 0.5	4.2 ± 0.6
$P_{e/c}$ Activity [µV]	-0.8 ± 0.6	2.1 ± 0.7	-0.7 ± 0.5	3.2 ± 0.6

For the $N_{e/c}$ amplitude the analyses of the untransformed ERPs showed a significant main effect of Accuracy, $F(1, 32) = 54.07$, $p < 0.001$, $\eta_p^2 = .63$, and of Self-evaluation, $F(1, 32) = 7.00$, $p = 0.013$, $\eta_p^2 = .18$, and a significant interaction of Accuracy and Self-evaluation, $F(1, 32) = 11.56$, $p = 0.002$, $\eta_p^2 = .27$. The N_e amplitude was larger for errors than for correct responses in the no-self-evaluation condition and in the self-evaluation condition, both p-values < 0.001. The $N_{e/c}$ amplitude for errors was smaller in the self-evaluation condition than in the no-self-evaluation condition, $p < 0.001$, while it did not differ significantly for correct responses, $p = 0.989$. Similar to the CSD-transformed data we only observed a significant effect of Accuracy for the $P_{e/c}$ amplitude, $F(1, 32) = 37.39$, $p < 0.001$, $\eta_p^2 = .54$, while there was no significant effect of Self-evaluation, $F(1, 32) = 0.87$, $p = 0.357$, $\eta_p^2 = .03$, and no significant Accuracy by Self-evaluation interaction, $F(1, 32) = 2.36$, $p = 0.135$, $\eta_p^2 = .07$. For the $P_{e/c}$ mean activity we find a significant effect of Accuracy, $F(1, 32) = 26.54$, $p < 0.001$, $\eta_p^2 = .45$, but no significant main effect of Self-evaluation, $F(1, 32) = 3.37$, $p = 0.076$, $\eta_p^2 = .10$. The interaction of Accuracy and Self-evaluation does not reach significance, $F(1, 32) = 2.82$, $p = 0.103$, $\eta_p^2 = .08$. The untransformed grand average waveforms are depicted in Figure 2.

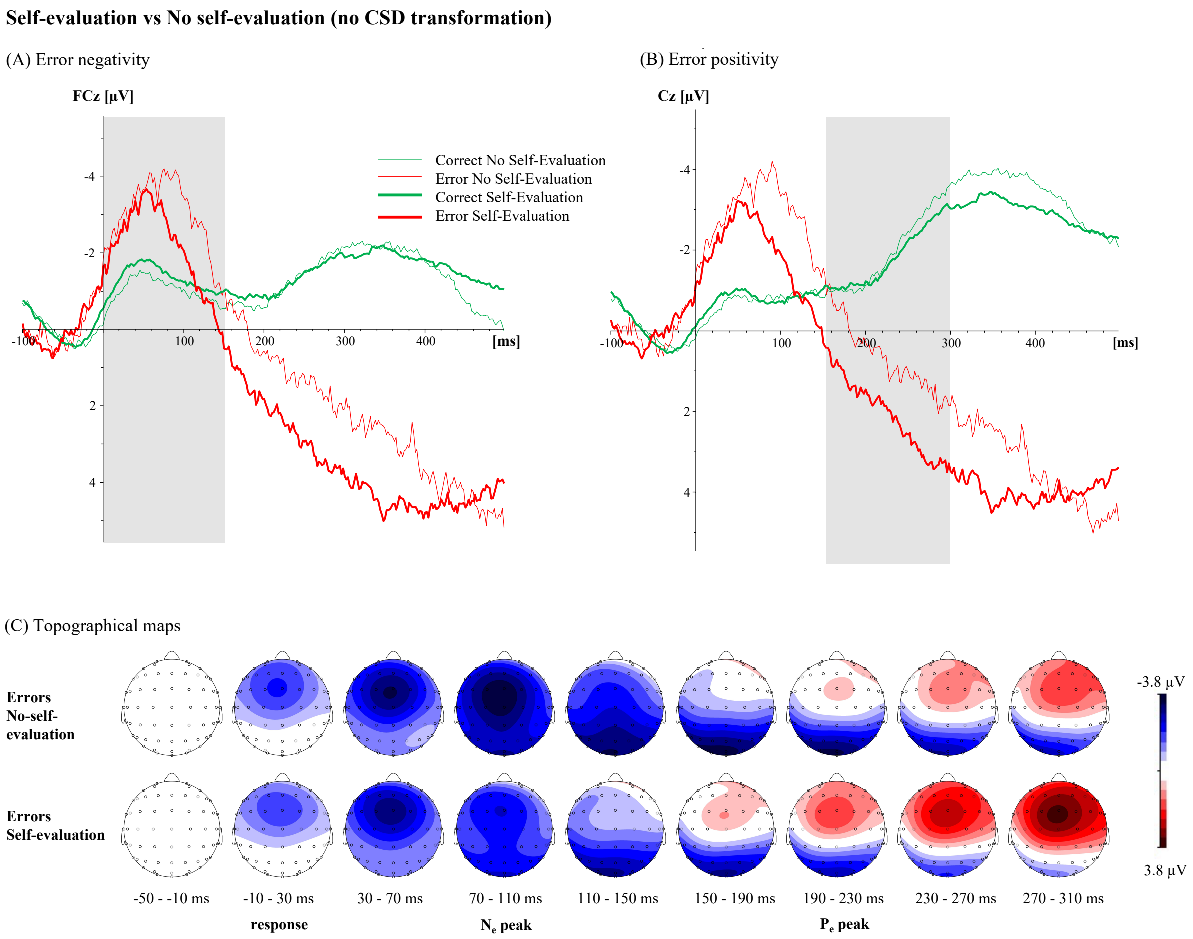

Figure 2. Averaged *un*transformed waveforms of the ERP component (A) error negativity, measured at FCz electrode as the mean amplitude (± 2 data points) around the negative peak in the time window of 0-150 ms after response onset and (B) error positivity, measured at Cz electrode as the mean amplitude (± 2 data points) around the positive peak and as mean activity in the time window of 150-300 ms after response onset grouped by accuracy (correct, error) and experimental condition (no-self-evaluation, self-evaluation), as well as the respective topographical maps for errors of both conditions.

Feature weights analyses

We conducted feature weights analyses to investigate from which channels the classifier uses information to decode the conditions from brain activity. We computed feature weights for the contrast between correct responses of the no-self-evaluation condition and correct responses of the self-evaluation condition, as well as for the contrast between errors from both conditions, as for these two contrasts the classifier was successful in decoding the condition from brain activity. We computed the feature weights for the time steps where the classifier decoded the condition significantly above chance (time steps 45 to 90 for correct responses and time steps 40 to 90 for errors). First, for each channel (i.e., each feature) the absolute feature weights were averaged within each of the 10 ms time steps. The resulting values were z-standardized for each time step. The significance of the feature

weights of each channel and each time step was tested with a series of Bonferroni-corrected *t*-tests. A significant feature weight indicates that the channel substantially contributes to the decoding accuracy of the classifier. The results of the feature weights analyses are depicted in Figure 3.

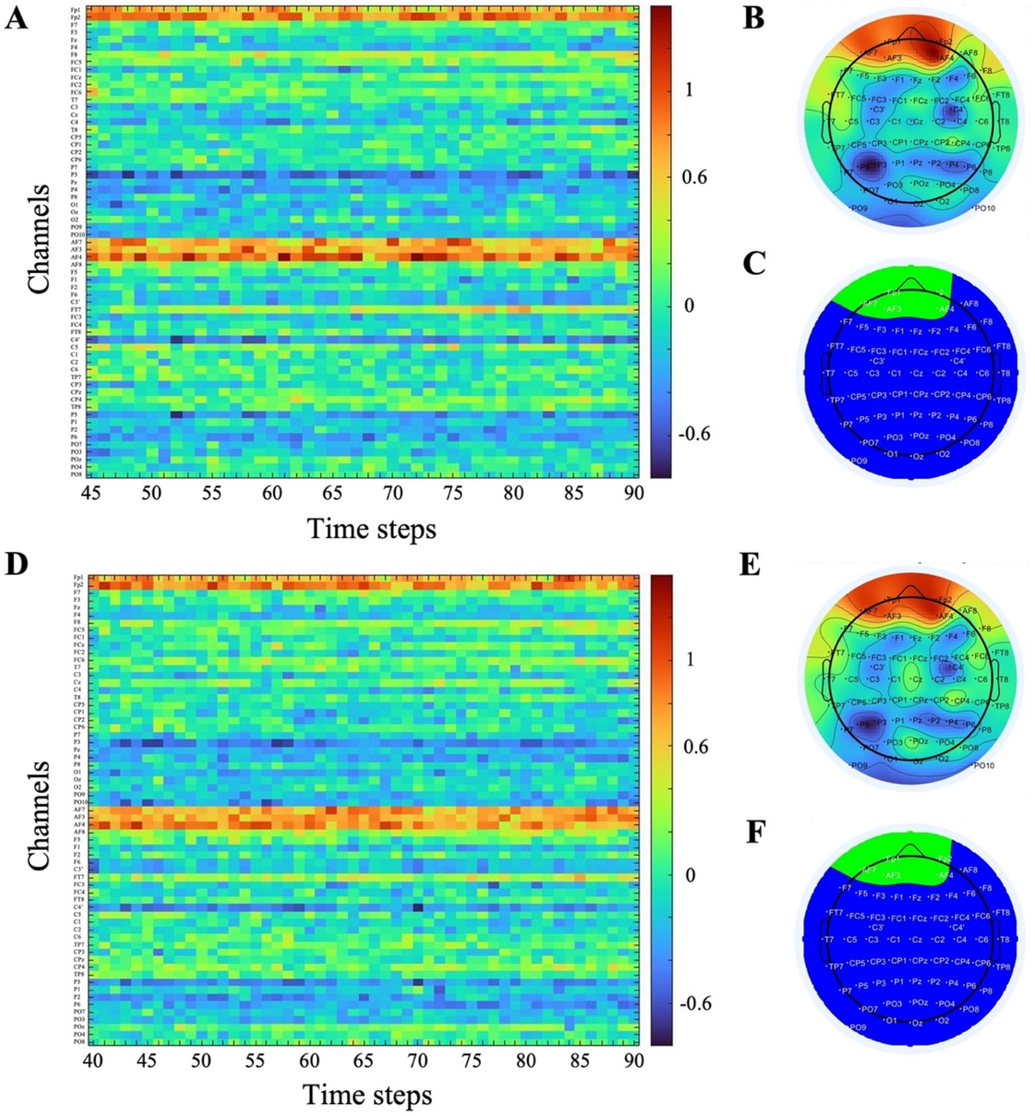

Figure 3. Z-standardized absolute feature weights averaged across the significant pre-response time steps (A and D) with the respective topographical maps (B and E) and the statistical threshold maps where significant features are color-coded green (C and F) for the contrast of correct responses of the no-self-evaluation condition vs correct responses of the self-evaluation condition (A-C) and for the contrast of errors of the no-self-evaluation condition vs errors of the self-evaluation condition (D-F).

The z-standardized absolute feature weights averaged across time steps show similar patterns for the contrast of the no-self-evaluation and the self-evaluation condition for both correct responses and errors. This suggests that the decoding success is not based on error specific processes. Significant features comprise anterior-frontal and fronto-parietal electrode sites. This is in line with the assumption that the MVPA results capture differences in broader cognitive control processes and

executive functions between the two self-evaluation conditions. However, this interpretation has to be made with care, as the distribution of the signal on the scalp is only an approximation for the underlying neural generators. Moreover, significant features can also be important because they systematically bind irrelevant variance, thus suppressing noise for other features that supply meaningful information (Haufe et al., 2014).

We also computed feature weights for the partwise MVPA contrasts. The results are depicted in Figure 4. Again, the results suggest that for all contrasts similar channels are important.

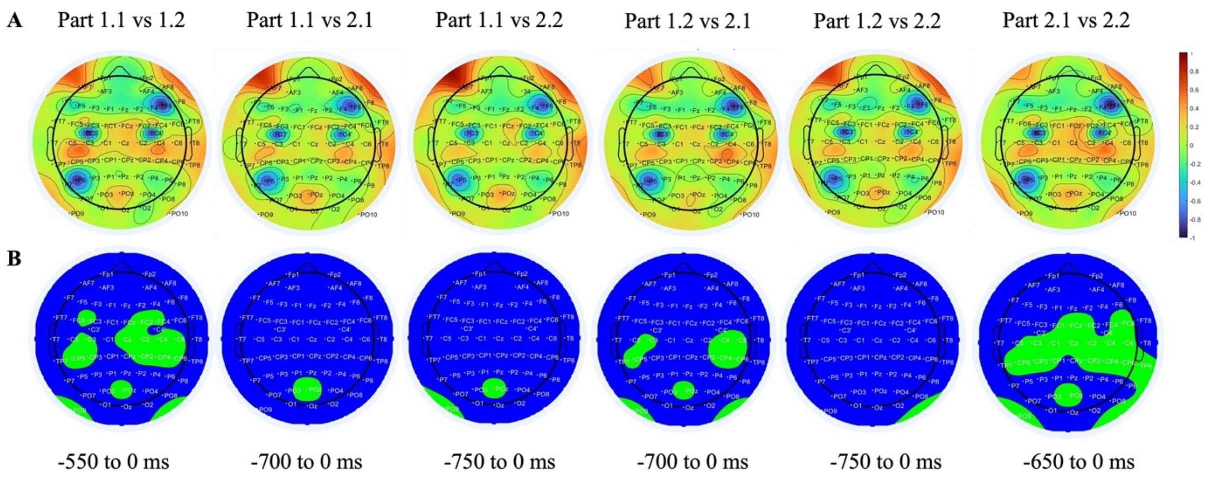

Figure 4. Z-standardized absolute feature weights averaged across the significant pre-response time steps mapped onto the scalp (A) and the respective statistical threshold maps where significant features are color-coded green (B) for the partwise contrasts of responses of the no-self-evaluation condition vs responses of the self-evaluation condition.

Trial-matched event-related potential results

The no-self-evaluation condition comprises less trials (320 trials) than the self-evaluation condition (704 trials). To ensure that the measurement of the N_e in the shorter no-self-evaluation condition is as stable as in the longer self-evaluation condition, we trial-matched the four conditions (errors and correct responses from the no-self-evaluation condition and the self-evaluation condition) based on the condition with the smallest number of trials (separately for each participant) and randomly drew the same number of trials from the other three conditions. We computed our ERP analyses for this trial-matched sample. The descriptive statistics are depicted in Table 3.

Table 3
Means ± standard error of means of the N_e amplitude (no-self-evaluation vs self-evaluation) for correct and erroneous responses for the non-trial-matched and the trial-matched data.

	**No-self-evaluation**		**Self-evaluation**	
	**Correct**	**Error**	**Correct**	**Error**
Non-trial-matched $N_{e/c}$ amplitude [µV/cm²]	-0.12 ± 0.02	-0.20 ± 0.03	-0.13 ± 0.02	-0.16 ± 0.02
Trial-matched $N_{e/c}$ amplitude [µV/cm²]	-0.15 ± 0.02	-0.20 ± 0.03	-0.18 ± 0.03	-0.17 ± 0.03

The trial-matched N_e amplitude and the non-trial-matched N_e amplitude did not differ significantly for errors in the no-self-evaluation condition, $t(32) = 1.84$, $p = 0.075$, $d = 0.32$, and for errors in the self-evaluation condition, $t(32) = 0.95$, $p = 0.349$, $d = 0.17$. We can thus rule out significant differences in N_e amplitude between the two conditions that are based on the number of trials.

Partwise Multivariate Pattern Analyses matched for response times

To investigate whether or not the results of the partwise MVPA are influenced by the change in response times across the experiment, we conducted RT-matched partwise MVPA. In RT-matched analyses, for the first-level MVPA only trials with similar response times are included in the contrasts. The results from the RT-matched partwise MVPA are depicted in Figure 5.

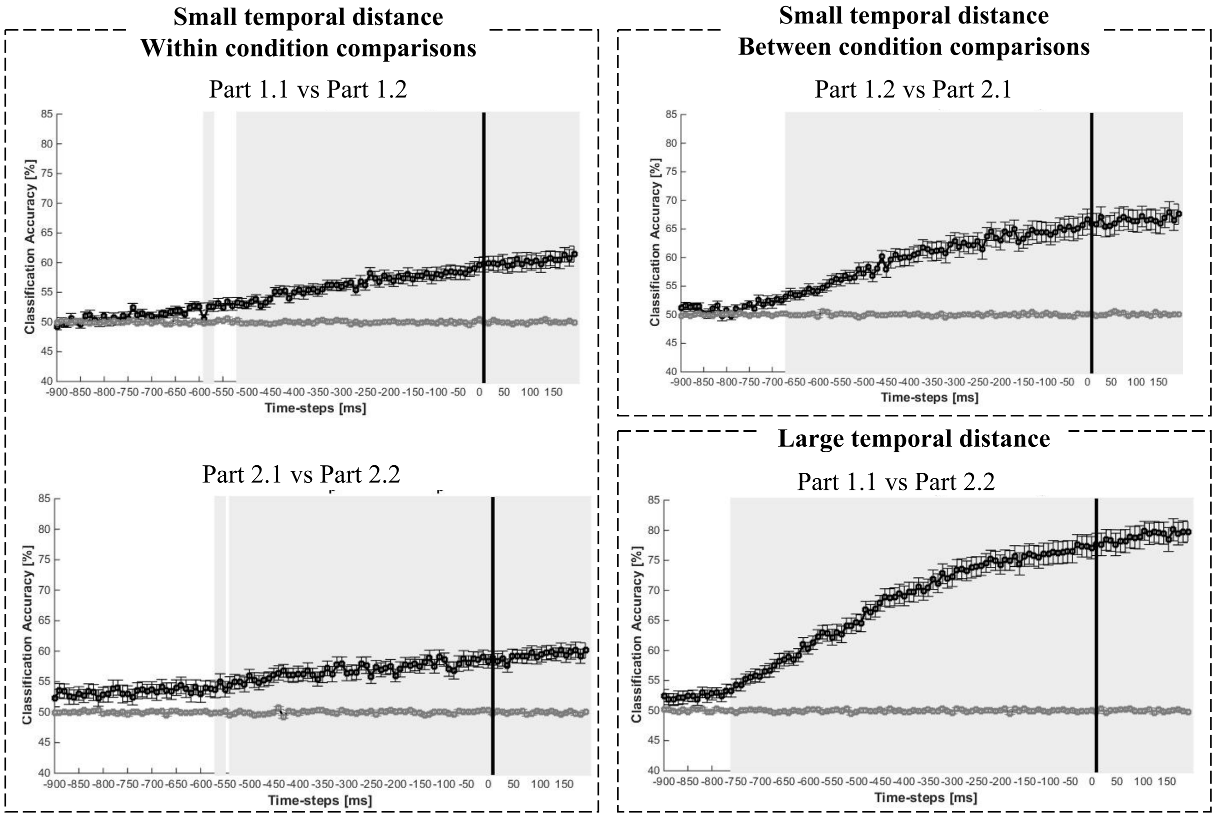

Figure 5. Classification accuracies in percent for each time step for RT-matched comparisons between the parts with small temporal distance within conditions (part 1.1 vs part 1.2 and part 2.1 vs part 2.2), with small temporal distance between conditions (part 1.2 vs part 2.1) and with the largest temporal distance (part 1.1 vs part 2.2). Grey areas indicate time windows in which the classification accuracies (black lines) differ significantly from the empirical chance level (grey lines).

The results show that the classifier obtains similar decoding accuracies for the partwise contrasts when the trials are RT-matched. As for the non-RT-matched data, the ANOVA for RT-matched data shows significant variations in the aggregated accuracy scores with Decoding Group, $F(2.24, 71.63) = 48.03$, $p < 0.001$, $\varepsilon = .45$, $\eta_p^2 = .60$. The accuracy scores were higher for the cross-condition decoding group with small temporal distance (part 1.2 vs 2.1, $M = 9.7$ %, $SE = 1.1$%) compared to both within-condition decoding groups with small temporal distances (part 1.1 vs 1.2, 5.3 ± 0.6%; and part 2.1 vs part 2.2, 6.2 ± 1.0%), $p < 0.001$ and $p = 0.014$, respectively. The within-condition decoding groups did not differ significantly, $p = 0.954$. The scores were highest for the decoding group contrasting the temporally most distant parts (part 1.1 vs part 2.2, 18.1 ± 1.2%), followed by the decoding groups contrasting parts of medium temporal distance (part 1.1 vs 2.1, 14.1 ± 1.3% and part 1.2 vs part 2.2, 14.6 ± 1.1), and then the decoding groups comparing parts of the smallest temporal distances, all p values < 0.014. The two decoding groups that compare parts of

medium temporal distance did not differ, $p = 0.997$. These results suggest that the behavioral differences in RT do not distort the partwise MVPA results.

Effects of time on task and counterbalancing

As the no-self-evaluation condition always preceded the self-evaluation condition, we cannot fully disentangle time on task effects from effects of self-evaluation. Unfortunately, the typical method to address time on task effects, counterbalancing, would not have resolved this problem. In a counterbalanced design the self-evaluation condition would precede the no-self-evaluation condition for half of the participants. In this order, for the preceding self-evaluation condition we could find a smaller N_e amplitude, a larger N_e amplitude or no difference in N_e amplitude between the two conditions. At first glance, a smaller N_e amplitude in the preceding self-evaluation condition (i) would be in line with our not counterbalanced results and thus suggest that in the self-evaluation condition less resources are available for fast error monitoring as effects of habituation and fatigue are controlled. However, in this case an N_e increase from the self-evaluation condition to the no-self-evaluation condition could also be explained by a (functional) carry-over effect from the self-evaluation condition to the no-self-evaluation condition (after several hundred times of self-evaluation it seems hard to not evaluate the performance even when one is not explicitly instructed to do so), by practice (which could lead to larger N_e amplitudes due to a more refined error monitoring process), and by a lowered error expectancy (which leads to larger N_e amplitudes according to the reinforcement learning theory; Holroyd & Coles, 2002). A larger N_e amplitude in the preceding self-evaluation condition (ii) would support the error significance account, but the N_e decrease towards the no-self-evaluation condition could also be an effect of habituation and fatigue. When no difference in N_e amplitude between the two conditions would be found (iii), effects of error significance, withdrawal of attentional resources, practice, habituation, and fatigue might all be at play and cancel each other out. These scenarios underline that counterbalancing cannot help to disentangle time on task effects from the effect of interest in our study.

To investigate the effect of time on task on the $N_{e/c}$ amplitude, we split the two self-evaluation conditions into two parts and conducted partwise ERP analyses. As depicted in Figure 6 we can

observe an N_e decrease from the no-self-evaluation condition to the self-evaluation condition rather than a continuous decline in N_e amplitude across the entire experiment.

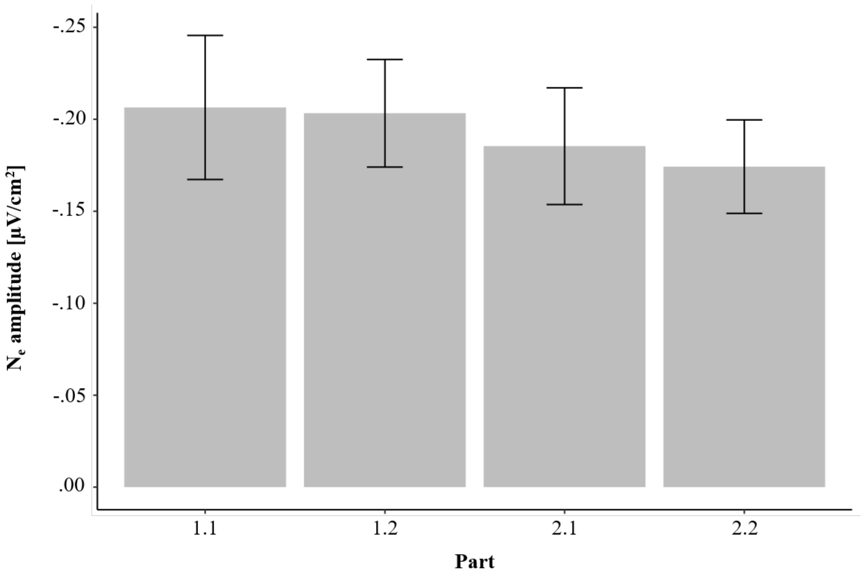

Figure 6. N_e amplitude (mean and standard error) for the four parts of the experiment for n = 29 participants. Data sets of four participants had to be excluded from the analyses as they contained less than six error trials per part after the artifact rejection.

The ANOVA with repeated measures for the factors Accuracy (error vs correct) and Part (1.1, 1.2, 2.1, 2.2) only shows a significant main effect of Accuracy on the $N_{e/c}$ amplitude, $F(1, 28) = 18.64$, $p < 0.001$, $\eta_p^2 = .40$, whereas Part, $F(2.45, 68.47) = 0.81$, $p = 0.472$, $\varepsilon = .82$, $\eta_p^2 = .03$, and the interaction of Accuracy and Part, $F(3, 84) = 1.96$, $p = 0.126$, $\varepsilon = .89$, $\eta_p^2 = .07$, did not show significant effects. Together, these findings suggest that the tendency for an N_e decrease from the no-self-evaluation condition to the self-evaluation condition captures an effect of self-evaluation rather than a mere time on task effect.

References

Haufe, S., Meinecke, F., Görgen, K., Dähne, S., Haynes, J.-D., Blankertz, B., & Bießmann, F. (2014). On the interpretation of weight vectors of linear models in multivariate neuroimaging. *NeuroImage*, *87*, 96–110. https://doi.org/10.1016/j.neuroimage.2013.10.067

Holroyd, C. B., & Coles, M. G. H. (2002). The neural basis of human error processing: Reinforcement learning, dopamine, and the error-related negativity. *Psychological Review*, *109*(4), 679–709. https://doi.org/10.1037/0033-295X.109.4.679
